# RollFISH achieves robust quantification of single-molecule RNA biomarkers in paraffin-embedded tumor tissue samples

**DOI:** 10.1038/s42003-018-0218-0

**Published:** 2018-11-28

**Authors:** Chenglin Wu, Michele Simonetti, Carla Rossell, Marco Mignardi, Reza Mirzazadeh, Laura Annaratone, Caterina Marchiò, Anna Sapino, Magda Bienko, Nicola Crosetto, Mats Nilsson

**Affiliations:** 1 Science for Life Laboratory, Department of Biochemistry and Biophysics, Stockholm University, Tomtebodavägen 23a, 17165 Solna, Stockholm Sweden; 2 Science for Life Laboratory, Department of Medical Biochemistry and Biophysics, Karolinska Institutet, Tomtebodavägen 23a, 17165 Solna, Stockholm Sweden; 3 Department of Medical Sciences, University of Turin, Corso Dogliotti 14, 10126 Turin, Italy; 4 Pathology Unit, Candiolo Cancer Institute—FPO-IRCCS, Strada Provinciale 142, 10060 Candiolo (TO), Italy; 5 Department of Immunology, Genetics and Pathology, Uppsala University, Dag Hammarskjölds väg 20, 75185 Uppsala, Sweden

**Keywords:** Fluorescence in situ hybridization, Breast cancer, Wide-field fluorescence microscopy, RNA probes

## Abstract

Single-molecule RNA fluorescence in situ hybridization (smFISH) represents a promising approach to quantify the expression of clinically useful biomarkers in tumor samples. However, routine application of smFISH to formalin-fixed, paraffin-embedded (FFPE) samples is challenging due to the low signal intensity and high background noise. Here we present RollFISH, a method combining the specificity of smFISH with the signal boosting of rolling circle amplification. We apply RollFISH to quantify widely used breast cancer biomarkers in cell lines and FFPE samples. Thanks to the high signal-to-noise ratio, we can visualize selected biomarkers at low magnification (20 × ) across entire tissue sections, and thus assess their spatial heterogeneity. Lastly, we apply RollFISH to quantify HER2 mRNA in 150 samples on a single tissue microarray, achieving a sensitivity and specificity of detection of HER2-positive samples of ~90%. RollFISH is a robust method for quantifying the expression and intratumor heterogeneity of biomarkers in FFPE tissues.

## Abstract (web summary)

Wu et al. introduce RollFISH, a method that enables quantification of single-molecule RNA with high specificity and sensitivity by combining smFISH with rolling circle amplification. RollFISH facilitated studying heterogeneity of biomarkers in formalin-fixed and paraffin-embedded breast cancer tissue, demonstrating its clinical application.

## Introduction

The advent of multi-region tumor sequencing has revealed that most cancer types harbor a high degree of intratumor heterogeneity^1,2^. This has profound implications on the analysis of molecular biomarkers for diagnostic, prognostic, and therapeutic purposes^1,2^. For example, a biomarker might be expressed at high levels within the primary tumor—which in most cases is the only site where a biopsy is taken—but at much lower or absent levels in one or more metastatic lesions. Furthermore, the fraction of tumor cells expressing the biomarker may vary from patient to patient. Additionally, the spatial distribution of cells expressing the biomarker might also represent a prognostic and/or predictive factor. Therefore, it is essential to develop quantitative methods that can reflect not only the abundance, but also the spatial distribution and heterogeneity of clinically relevant biomarkers inside tumor samples.

In situ RNA fluorescence hybridization assays represent a promising approach to quantify clinically relevant biomarkers in tumor specimens^3^. In particular, single-molecule RNA FISH (smFISH) is a versatile assay that allows detecting single RNA molecules with high specificity^4^. In smFISH, a set of typically 30–50 oligonucleotides of 20 nucleotides (nt), each conjugated to a single fluorophore, are firstly hybridized to a complementary RNA target. Individual transcripts are then visualized as diffraction-limited spots using wide-field epifluorescence microscopy, and quantified^5^. We recently applied smFISH to quantify the expression and topographic distribution of two prominent breast cancer biomarkers and drug targets, epidermal growth factor receptor 2 (HER2) and estrogen receptor 1 (ER) in FFPE samples^6^. A major limitation of this approach, however, is that smFISH signals are dim, while background fluorescence is high in FFPE samples (with large variability depending on the tissue type, sample age, and fixation conditions). Hence, imaging at high magnification (60–100 × ) is required. As a result, only a very small area of the sample is usually imaged (typically, 40–50 fields of view), making it difficult to assess intratumor heterogeneity.

In contrast to smFISH, other methods such as RNAscope^7^, rolling circle amplification (RCA) of padlock probes^8,9^, and single-molecule hybridization chain reaction (smHCR)^10^ involve one or more steps of signal amplification, which results in brighter fluorescence signals and higher signal-to-noise ratio. However, all of these methods have a number of drawbacks that limit their utility for the purpose of quantifying the intratumor heterogeneity of clinically relevant biomarkers. The sensitivity of RNAscope is lower compared to smFISH. Limited sensitivity is also a major drawback of padlock probe and RCA, due to the fact that RNA needs first to be converted to cDNA in situ, which is inefficient. A recent study reported direct detection of mRNA in single cells based on RCA, however, the detection efficiency remains low^11^. Fluorescent in situ SEQuencing (FISSEQ) is another approach in which RCA products (RCPs) are generated from self-circularized cDNA in a non-targeted fashion, and then sequenced in situ by SOLiD technology^12^. Similar to conventional RCA, the sensitivity of FISSEQ is low and the method is biased towards abundant transcripts. Lastly, smHCR is limited by low specificity due to the fact that smHCR hairpins can bind nonspecifically within the sample and become subsequently amplified^13,14^. To destabilize nonspecific binding, smHCR is typically performed under very stringent hybridization conditions, which however limit the number of RNA molecules that can be effectively detected^13,14^.

In this study, we aim to overcome the above limitations by combining the versatility and sensitivity of smFISH with the signal amplification power of RCA. Using this combined RCA-smFISH (RollFISH) approach, we visualize and accurately quantify clinically relevant biomarkers, at single-cell resolution, across entire FFPE tissue sections. We conclude that RollFISH is a robust method that can be readily adapted to quantify the spatial heterogeneity of clinical biomarkers in FFPE tissue samples.

## Results

### Implementation of RollFISH

**Fig. 1:**
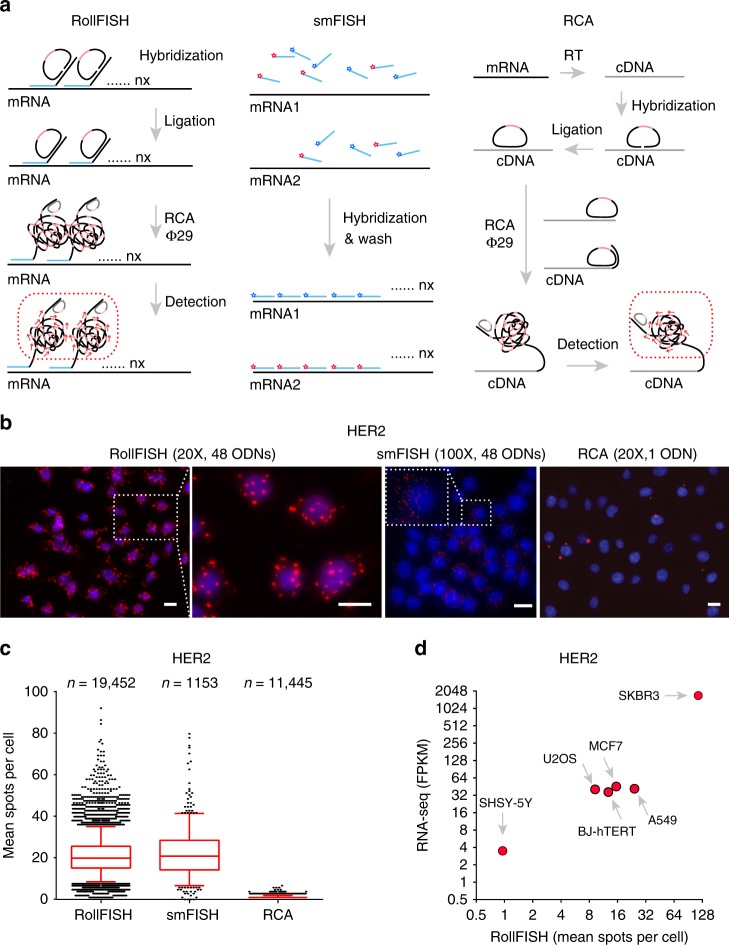
Implementation and validation of RollFISH. **a** Workflow of RollFISH in comparison to smFISH and standard RCA. Each ODN in a RollFISH probe consists of 30nt complementary to the target RNA sequence and of 46nt orthogonal to the human transcriptome, serving as docking sequence for a padlock probe. A hinge sequence of four thymidines (T) is included between the two sequences to facilitate recognition and binding of the padlock probe to the docking sequence. **b** Representative images of RollFISH, smFISH, and standard RCA for HER2 in A549 cells. The magnification used and the number of ODNs per probe is shown. Blue, Nuclei. Scale bar, 10 µm. **c** Quantification of the number of spots per cell in images of which those in **b** are representative. Boxes extend from the 25th to the 75th percentile. Whiskers extend from 2.5 to 97.5 percentiles. The line inside each box represents the median value. Three experiments were performed for each assay, and all the data were pooled into one box plot. *n*, total number of cells analyzed. **d** Comparison of HER2 levels detected in six different cell lines by RollFISH and RNA-seq. RollFISH was repeated twice and the mean value for each cell line is plotted

We modified the design of smFISH probes so that padlock probes can be docked to them, thus enabling signal amplification. A comparison of RollFISH, smFISH and standard RCA is shown in Fig. 1a. Briefly, each RollFISH probe consists of a set of oligodeoxynucleotides (ODNs), each containing a 30nt sequence complementary to the RNA target, followed by a 46nt docking sequence orthogonal to the human transcriptome on the 3′ end. The ODNs are firstly hybridized in situ to their complementary target, followed by removal of unspecifically bound ODNs. Next, a padlock probe containing a transcript-specific barcode sequence is docked to each ODN and circularized in situ to form a single-stranded DNA circle. As in standard RCA, rolling circle amplification is then carried out using the Phi29 polymerase primed by the 3′ end of the ODNs. The resulting RCA product (RCP) contains hundreds to thousands of copies of the reverse sequence of the transcript-specific barcode sequence, which is detected using a fluorescently labeled secondary ODN. Because a RollFISH probe consists of many ODNs, multiple RCPs can form simultaneously on the same transcript molecule, resulting in a bright fluorescent spot that can be visualized at low magnification (20 × ) using wide-field epifluorescence microscopy.

To optimize the duration of the RCA step in RollFISH, we designed a probe targeting the epidermal growth factor receptor 2/ERBB2/HER2 mRNA, and visualized the RCPs after 20, 40, 60, 80, and 100 min of RCA (Supplementary Fig. 1a and Supplementary Table 1). The number of detected spots per cell did not vary substantially by increasing the RCA time (17 ± 7 spots per cell at 20 min; 24 ± 9 spots per cell at 100 min), while the signal-to-noise ratio increased more than two-fold (Supplementary Fig. 1b, c). We found that the optimal duration of RCA is about 60 min, preventing RCPs to become too large and merge with RCPs from adjacent transcripts.

Next, we determined how many ODNs per probe are needed to detect HER2 transcripts in comparison to smFISH. To this end, we created a series of RollFISH probes containing one up to 48 ODNs complementary to HER2 mRNA (Supplementary Fig. 2a). The number of detected spots per cell sharply increased from one ODN to 12 ODNs per probe, while it increased considerably less by doubling the number of ODNs from 24 to 48, which is the typical number of ODNs per probe used in smFISH (Supplementary Fig. 2b). As expected, there were more spots with higher signal intensity when a higher number of ODNs were used (Supplementary Fig. 2c). On average, the intensity of RollFISH fluorescent spots with 48 ODNs per probe was 25% higher than the intensity of the signals with one ODN per probe. This difference reflects the different number of RCPs that are formed on the same RNA molecule, by using a different amount of ODNs per probe. To assess the detection efficiency of RollFISH, we performed an experiment in which we used two padlock probes in two different colors to detect the same RollFISH probe (Supplementary Fig. 3a, b). The colocalization between the two colors stably increased with the number of ODNs per probe, reaching approximately 50% at 48 ODNs per probe, which corresponds to a detection efficiency of ~70% (Supplementary Fig. 3b, c). This is very similar to the detection efficiency (67.5%) previously reported for smFISH using a similar colocalization assay^15^. These results indicate that RollFISH is able to efficiently detect RNA transcripts in single cells using a relatively short RCA time and a lower number of ODNs per probe, compared to smFISH.

### Validation of RollFISH

Next, we sought to validate RollFISH by comparing it to existing methods for RNA quantification in situ or in vitro. First, we assessed the sensitivity of RollFISH by comparing HER2 transcript counts measured by RollFISH, smFISH, or standard RCA (Fig. 1b, c). While both RollFISH and smFISH detected many HER2 transcripts at comparable levels (median: 21, range: 1–97 spots per cell for RollFISH; median: 26, range: 1–83 spots per cell for smFISH), standard RCA only managed to detect a few transcripts (Fig. 1b, c). We then correlated RollFISH HER2 transcript counts with available RNA-seq data for six different cell lines. There was a significant positive correlation (Pearson’s *R*^2^ = 0.97) between the number of HER2 transcripts per cell detected by RollFISH and the corresponding number of fragments per kilobase per million mapped reads (FPKM) measured by RNA-seq (Fig. 1d). We also designed probes targeting ER and MKI67/Ki-67 transcripts (Supplementary Table 1), so that each target gene can be visualized in a different color. Quantification of these genes together with HER2 in six different cell lines showed cell-specific expression patterns (Supplementary Fig. 4a, b). Additionally, the ER and Ki-67 RollFISH counts scaled with RNA-seq FPKM values, further confirming the specificity of RollFISH (Supplementary Fig. 4c, d). Altogether, these results demonstrate that RollFISH enables accurate detection and quantification of individual RNA transcripts in single cells in situ.

### Heterogeneity revealed by RollFISH in FFPE breast cancer tissues

**Fig. 2:**
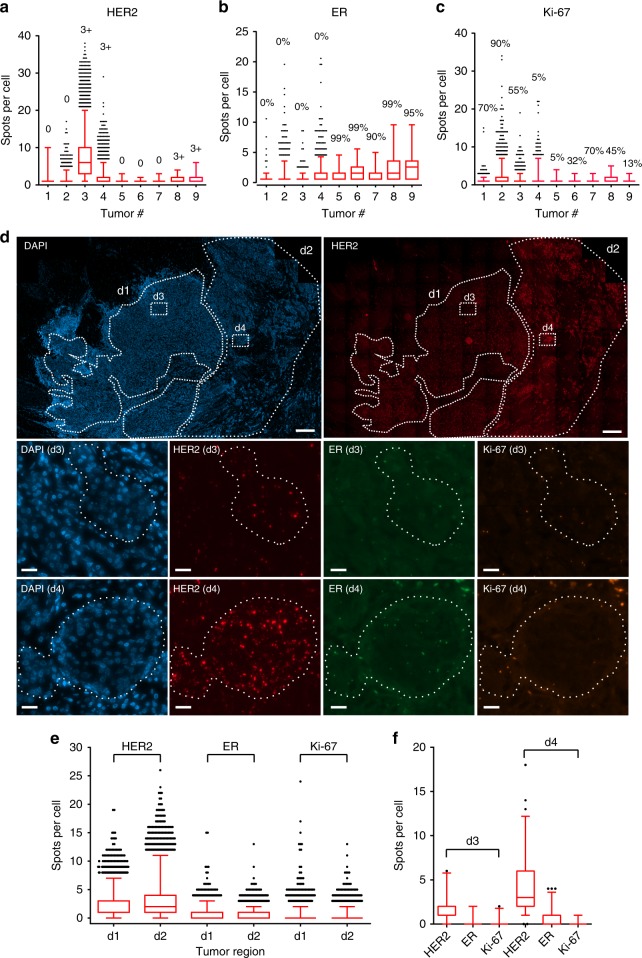
RollFISH in FFPE breast cancer samples. **a–c** Expression of HER2, ER, and Ki-67 in breast cancer tissues. Pathological information for each of the samples shown is listed in Supplementary Tables 2 and 3. The percentages above each column in **b** and **c** indicate the proportion of positive tumor cells as revealed by IHC. **d** The expression of HER2, ER, and Ki-67 across an entire FFPE tissue section of tumor #8 (see Supplementary Tables 2 and 3). The regions in dashed boxes (d3 and d4) in upper large images (first-row) are zoomed-in, and shown below as two rows of images with region names (d3 and d4). Scale bars, 200 µm (first-row images) and 30 µm (second- and third-row images). Blue, Nuclei. **e** Quantification of HER2, ER, and Ki-67 in regions d1 and d2 in **d**. Cells without any signals were excluded from the analysis. **f** Expression of HER2, ER, and Ki-67 in regions d3 and d4. In all the box plots presented in this figure, boxes extend from the 25th to the 75th percentile, whiskers extend from 2.5 to 97.5 percentiles, and the line inside each box represents the median value

We then applied RollFISH to FFPE breast cancer tissue samples. We quantified HER2, ER, and Ki-67 transcripts in nine breast cancer samples, including all major molecular subtypes (Supplementary Table 2). Numerous RollFISH spots could be readily detected in tumor regions densely populated by tumor cells, but not in the adjacent stroma (Supplementary Fig. 5). To confirm the specificity of RollFISH probes in FFPE tissue, we performed automatic segmentation and classification of thousands of cells, and counted the number of HER2 transcripts inside tumor vs. non-tumor cells, as well as in tissue areas without cells, in one sample (tumor #8) (Supplementary Fig. 6). 90% of the transcripts colocalized with cancer cells, while only 6% of them were detected in non-cellulated stromal areas, which is indicative of high specificity (Supplementary Fig. 6b, d). RollFISH counts matched well with pathological reports of the same markers previously assessed by immunohistochemistry (IHC) (Fig. 2a–c and Supplementary Table 2 and 3). In the case of HER2, distinct RollFISH spots were observed in both HER2 + and luminal B/HER2 + tissues, while a much lower signal density was observed in triple negative, luminal A, and luminal B/HER2– subtypes. The expression of Ki-67 in some of the tissues did not match well with the respective pathological reports, which might be caused by low transcript abundance (Fig. 2c). Indeed, an analysis of RNA-seq data from more than one thousand breast cancers in The Human Protein Atlas showed that Ki-67 transcripts are typically expressed at low levels (Supplementary Fig. 7). Moreover, it is also well known that conventional histopathological Ki-67 can be challenging and poorly reproducible even among expert pathologists^16^.

The ability of visualizing RollFISH signals across the entire tissue section allowed us to assess the extent of spatial heterogeneity of HER2, ER, and Ki-67, in a way that would not be possible with smFISH. For example, in tissue #8, two large regions within the same FFPE tissue section showed very different levels of HER2 expression, whereas ER and Ki-67 levels were similar between the same two regions (Fig. 2d, e). Inside these regions, there was considerable cell-to-cell variability in the expression levels of the three genes (Fig. 2d, f). Retrospective evaluation of the pathological report of HER2 IHC confirmed the presence of heterogeneity of HER2 expression, with two distinct tumor cell populations with HER2 score 1 + and 3 + , respectively, coexisting within the same tissue section (Supplementary Fig. 8). Overall, the above results demonstrate that RollFISH can be used to quantify both the abundance and the spatial heterogeneity of RNA biomarkers in FFPE breast cancer tissue.

**Fig. 3:**
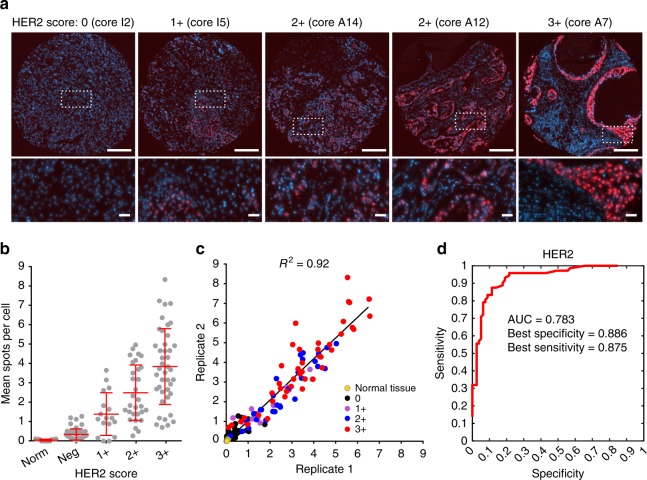
RollFISH in tissue microarrays. **a** Examples of HER2 levels in five different cores. Regions inside dashed boxes in first-row images are magnified below. Core names correspond to the position of the core in the TMA, as shown in Supplementary Fig. 9. Blue, Nuclei. Scale bar, 200 µm (first-row images) or 25 µm (second row images). **b** HER2 levels by IHC group in one replicate experiment. Each dot corresponds to one sample. Error bars show the mean value ± s.d. for each IHC score group. Norm, normal breast tissues or adrenal gland. Neg, 0 for IHC HER2 score. **c** Correlation between RollFISH counts in two replicate experiments on two consecutive TMA sections. The black line represents the best linear regression fit. *R*^2^, Pearson’s correlation coefficient. Each dot represents a sample and samples were color-coded based on their HER2 IHC score. **d** Receiver operating characteristic curve of the sensitivity and specificity with which RollFISH correctly detects HER2-positive tumors previously classified based on IHC

Lastly, we aimed to demonstrate the applicability of RollFISH to a large number of FFPE samples in parallel. To this end, we applied the HER2 RollFISH probe validated above (consisting of 48 ODNs) to detect HER2 transcripts in 75 samples represented in duplicate on a single tissue microarray (TMA), including normal breast tissue as well as breast cancers with HER2 IHC score ranging from 0 to 3 + (Supplementary Fig. 9a, b, Supplementary Tables 4 and 5, and Methods). In samples belonging to the 2 + and 3 + groups, RollFISH detected HER2 transcripts in high abundance, whereas HER2 was expressed at low levels or absent in 0 and 1 + tumors, as well as in normal tissues (Fig. 3a, b). Low RollFISH counts were found in only 4 out of 42 samples (9.5%) with IHC 3 + (Supplementary Fig. 9b). mRNA degradation might have occurred in these samples, similar to what has been recently reported^17^. In two independent experiments conducted in two consecutive sections of the same TMA, the results of RollFISH were highly reproducible (Pearson’s *R*^2^ = 0.92, *p*-value < 0.0001, Fig. 3c). Finally, we used these data to assess the sensitivity and specificity of RollFISH using the receiver operating characteristic curve method (Methods). RollFISH was able to distinguish HER2-positive (2 + and 3 + ) from HER2-negative (0 and 1 + ) samples with a sensitivity and specificity of 88.6% and 87.5%, respectively (Fig. 3d). These results indicate that RollFISH can be applied for high-throughput screening of potentially useful biomarkers in tumor TMAs.

## Discussion

We have developed a versatile assay that integrates the specificity of smFISH with the signal amplification capability of RCA to quantify transcript biomarkers across entire FFPE tissue sections. Key advantages of RollFISH include: (1) streamlined probe design and preparation; (2) use of unlabeled gene-specific probes and the requirement for a smaller number of ODNs per probe compared to smFISH, which makes the method more cost-effective; (3) ability to image transcripts over large portions of a tissue section at low magnification (20 × ), which is more common in pathology laboratories; (4) applicability to archival FFPE tissue sections and aged FFPE samples in which smFISH and other fluorescence-based RNA detection methods are notoriously challenging; (5) applicability to TMAs for high-throughput screening of potentially useful biomarkers. These features make RollFISH a powerful method that can be readily integrated in routine pathology diagnostics to quantify the abundance and chart the topography of clinical biomarkers in FFPE samples.

In this study, we have focused on clinical biomarkers in FFPE samples. However, applications of RollFISH are not limited to biomarker detection in FFPE samples. For example, in neuroscience, an area of active research focuses on using single-cell RNA-seq to comprehensively identify all cellular subtypes in the brain, with the ultimate goal of achieving an exhaustive map of the cellular network that supports high-level cognitive functions. RollFISH could be used to identify the spatial location of newly predicted cellular subtypes based on the detection of a set of transcripts that specifically mark a given cell subtype. High-throughput spatial transcriptomic techniques, including sequential smFISH^18^, spatial transcriptomics^19^ and MERFISH^20^, have also been applied to map cellular diversity in human and mouse brain^21^. However, these methods are technically challenging and still require posterior validation by lower-throughput assays such as smFISH. In this context, RollFISH could prove very valuable especially when samples characterized by high levels of tissue auto-fluorescence need to be analyzed (e.g., aged brain).

Another potential application of RollFISH is high-throughput screening. For example, in drug screening, RollFISH could be used to detect specific transcripts that serve as markers of drug activity. As we have shown in this study, RollFISH can also be used to screen the expression of selected biomarkers across hundreds of tumor samples in parallel on TMAs. Finally, as we were able to detect a bright signal already with 4–5 ODNs per probe, RollFISH could be applied to detect short RNAs or to discriminate between different splicing variants, which is not possible by smFISH. In conclusion, RollFISH is a versatile, scalable and cost-effective method that can be used to quantify RNA molecules also in challenging FFPE samples, with broad applications ranging from research to routine diagnostics.

## Methods

### Cell culture

BJ-hTERT and MCF7 cells were cultured in Dulbecco’s modification of Eagle medium (DMEM) (Gibco, cat. no. 11960044), U2OS and SKBR3 cells in McCoy’s 5a medium (Sigma, cat. no. M4892), A549 cells in RPMI 1640 (Sigma, cat. no. R0883), SHSY-5Y cells in a 1:1 mixture of Ham’s F12 (Gibco, cat. no. 11765054) and DMEM medium (Gibco). All media were supplemented with l-glutamine and phenol red, 2mM l-glutamine (Sigma, cat. no. 59202C), 10% FBS (Sigma, cat. no. F7524) and 1 × PEST (Sigma, cat. no. P4333). All cell lines were incubated at 37 °C in 5% CO_2_. To prepare samples for RollFISH, cells were treated with 0.25% (w/v) trypsin-EDTA (Sigma, cat. no. T4049) and resuspended in culturing medium. Suspended cells were seeded on five Superfrost Plus slides (Thermo Fisher Scientific, cat. no. J1800AMNT) placed in a 150 × 25 mm Petri dish (Corning, cat. no. CLS430597), and culturing medium was added to a final volume of 25 ml. Cells were incubated for 12 h in the same previous culture conditions. Fixation was then performed in 3.7% (w/v) paraformaldehyde (Sigma, cat. no. P6148) in DEPC-treated PBS for 20 min at room temperature. After fixation, slides were washed twice in DEPC-treated PBS and dehydrated in an ethanol series of 70%, 85% and 100% for 5 min each. Cell slides were kept at −80 °C until use. Secure-Seal hybridization chambers (Grace Bio-Labs, cat. no. PC50) were used for following experiments.

### FFPE tissues

Formalin-fixed, paraffin-embedded (FFPE) tissue sections (4μm thick) of human breast cancer tissues were obtained from the Pathology Division, Department of Medical Sciences, University of Turin, Italy (Supplementary Table 2). FFPE tissues were stored at room temperature after received. The study was conducted under ethical permission granted by the Committee for human Biospecimen Utilization (DSM-ChBU) of the Department of Medical Sciences, University of Turin, Italy. Written informed consent was obtained from all cancer patients for collection, storage, and research use of both fresh and archival tumor samples that were anonymized with alphanumeric code. The samples were stored under vacuum at + 4 °C until sampling and fixation. Positivity for hormone receptors (ER and PR) and HER2 was assessed following the ASCO/CAP guidelines^22,23^, and the percentage of Ki-67 positive cells was reported according to international recommendations^24^. Molecular subgroups were assigned based on the IHC surrogate proposed by the St. Gallen International Expert Consensus^25^. FFPE tissue microarray sections (5μm thick) were obtained from US Biomax, Inc. The array consisted of 150 cores (75 cases in duplicates) of breast cancer tissues and adjacent normal tissues and the diameter of each core was 1 mm. Information on pathological grade, clinical stage and IHC evaluation of ER, PR, and HER2 was available for each sample (Supplementary Fig. 9a and Supplementary Tables 4 and 5). The samples were stored at + 4 °C before experiments. In preparation for RollFISH, all FFPE samples were deparaffinized and processed as previously described^6^. In the case of TMAs, the tissue sections were baked at 60 °C for 2 h, deparaffinized in xylene and rehydrated in an ethanol series. RNA retrieval was carried out at 95 °C for 15 min. The sections were then covered with Secure-Seal hybridization chambers (EMS, cat.no. 70333–22) before proceeding to the subsequent steps. All the steps were performed in special plastic jars (EMS, cat. no 71385) decontaminated with RNaseZAP (Ambion, cat. no AM9780), and all the solutions were prepared in RNase-free water (Ambion, cat. no. AM9932).

### smFISH

smFISH probes targeting the coding sequence of *HER2*, *ER* and *Ki-67* genes were designed based on the previously described database of 20-mers covering all human transcripts (www.fusefish.eu^26^). The complete list of sequences of the ODNs constituting each probe is provided in Supplementary Table 1. Hybridization was performed as previously described^6^. Data plotted in Fig. 1c were generated during previous study^6^.

### RollFISH

All samples (both cells and tissues) were firstly incubated in a wash buffer containing 5 × SSC, 30% formamide and 0.1% Tween (Sigma, cat. no. P1379) for 10 min at room temperature. The samples were then incubated overnight in a buffer containing 5 × SSC, 100 μg/ml salmon sperm DNA (Thermo, cat. no. 15632011), 0.25 mg/ml *E.coli* tRNA (Sigma, cat. no. R4251), 30% formamide (Thermo, cat. no. 17899), 2 mM RVC (NEB, cat. no. S1402S) and 0.1 μM of each probe. The samples were then washed three times in wash buffer for 10 min at room temperature. Next, a mix containing 1 × Ampligase buffer (20 mM Tris-HCl, pH 8.3, 25 mM KCl, 10 mM MgCl_2_, 0.5 mM NAD and 0.01% Triton X-100), 100 nM of each padlock probe, 50 μM dNTPs, 0.5U/μl Ampligase (Illumina, cat. no. A3210K), 50 mM KCl and 20% formamide was added into the Secure-Seal hybridization chambers (EMS, cat.no. 70333–22) mounted on the slides. Samples were incubated at 37 °C for 30 min, 45 °C for 45 min, and then washed twice in 1 × DEPC-PBS-T (1 × DEPC-treated PBS, 0.1% Tween 20, pH 7.4). The complete list of sequences of padlock probes and detection fluorescent oligonucleotides is provided in Supplementary Table 1. Finally, samples were incubated in RCA buffer containing 1U/μl phi29 polymerase (Thermo, cat. no. EP0091), 1 × phi29 polymerase buffer, 0.25 mM dNTPs (Thermo, cat. no. R0191), 0.2 μg/μl BSA (NEB cat. no. B9000S) and 5% glycerol in DEPC-H_2_O for 1 h or otherwise specified. Samples were then washed twice in DEPC-PBS-T, and incubated in a buffer containing 2 × SSC, 20% formamide, and 100 nM of the detection oligos at 37 °C for 30 min. Unbound detection oligos were removed with two DEPC-PBS-T washes.

### Standard RCA

Samples were firstly washed with DEPC-PBS-T twice. For cell line, Secure-Seal hybridization chambers were used directly on cell slide after an ethanol series of 70%, 85%, and 100% for 1 min each to remove water. The cell line was permeabilized in 0.1 M HCl at 37 °C for 1 min before two washes with DEPC-PBS-T. A reversed transcription mix, containing 500 μM dNTPs (Thermo), 5 μM of unmodified random decamers, 0.2 μg/μl BSA (NEB), 20U/μl of TranscriptMe reverse transcriptase (DNA Gdansk, cat. no. RT32), and 1U/μl RiboLock RNase Inhibitor (Thermo, cat. no. EO0381) in the TranscriptMe reaction buffer, was applied on the slides. The sequences of all padlock probes used in this study are listed in Supplementary Table 1. The incubation was carried out for 3 h at 37 °C for cell line and overnight (18 h) for tissue section. Slides were washed twice with DEPC-PBS-T, and followed by a post-fixation step in 3.7% (w/v) paraformaldehyde in DEPC-PBS for 30 min (5 min for cell line) at room temperature. After post-fixation, the samples were washed twice in DEPC-PBS-T. After reverse transcription, RNA degradation, hybridization and ligation of padlock probe and RCA on synthesized cDNA were performed as above in RollFISH section. RCA was carried out for 2.5 h for cell line and overnight (18 h) for tissue slide. After two washes with 1 × DEPC-PBS-T, RCA products were detected with 100 nM of fluorescence conjugated detection probes in 2 × SSC buffer with 20% formamide at 37 °C for 30 min.

### Image acquisition and analysis

For smFISH, images were acquired using an Eclipse Ti2 inverted microscope (Nikon) using a 100 × objective. For tissues that underwent RollFISH or standard RCA, images were acquired using an AxioplanII epifluorescence microscope (Zeiss). Image stacks were captured for RCPs located in different focal planes, and then merged into a single image using the maximum-intensity projection (MIP) in the Zeiss AxioVision software. Exposure times for all the experiments are listed in Supplementary Table 6. For tissue scans, a 10% overlap between two neighboring images was set. The resulting images were then automatically stitched together into a single image. The images for gene expression profiling in the entire scanned area were generated by using a 20 × objective. The stitched image was used for further image analysis. Briefly, images were firstly cropped to a region of interest in order to remove edge effects resulting from the MIP. Cell nuclei were separated based on shape descriptors. Definition of cell cytoplasm uses nucleus as a seed and depends on sufficient cytoplasmic auto-fluorescence, and once such an approach failed, cytoplasm was defined as region within a fixed distance from a nucleus. The image of the general stain from the firstly hybridization step was enhanced by a top-hat filter. Each RCP was given a unique label after separated by watershed segmentation. All steps were performed using CellProfiler (2.1.1, 6c2d896), and the results were saved as.csv files.

To automatically segment and classify cells in FFPE tissues, we firstly used Ilastik^27^, a pixel classifier software, to segment all the cells in the scan. We then transferred the obtained masks in MATLAB to define the borders of each cell. To discriminate tumor cells from other cells, we manually measured with ImageJ the nucleus area/perimeter ratio and roundness of 202 putative tumor cells stained with DAPI. We then subtracted three times the standard deviation from the median of the values, both for ratio (4.08) and roundness (0.46). To map the coordinates of RollFISH spots, we used custom-made scripts in MATLAB previously described^6,26^. Lastly, we overlaid the coordinates of the spots with boundaries of the segmented cells. We counted the number of spots falling inside or outside cells, after expanding the segmentation margin of 9.75 μm in all directions. We also counted the number of spots falling in tumor cells vs. non-tumor cells.

### Receiver operating characteristic curve analysis

We performed receiver operating characteristic curve analysis to assess the diagnostic specificity and sensitivity of RollFISH for HER2 as previously described^6^. Briefly, we binarized the IHC scores (Supplementary Table 5), and considered 2 + and 3 + cases as positive. We screened 100 cutoffs of the mean number of spots per cell, starting at 0.05 spots per cell in increments of the same magnitude.

## Electronic supplementary material

Supplementary Information

## Data Availability

The datasets generated in current study are available from the corresponding authors upon request.
