# Supplementary material for: RollFISH achieves robust quantification of single-molecule RNA biomarkers in paraffin-embedded tumor tissue samples
Source: Commun Biol. 2018 Nov 28;1:209. doi: 10.1038/s42003-018-0218-0 (PMC6262000; doi:10.1038/s42003-018-0218-0)
Supplement: Supplementary file 1 — Supplementary Information [file 42003_2018_218_MOESM1_ESM.pdf]

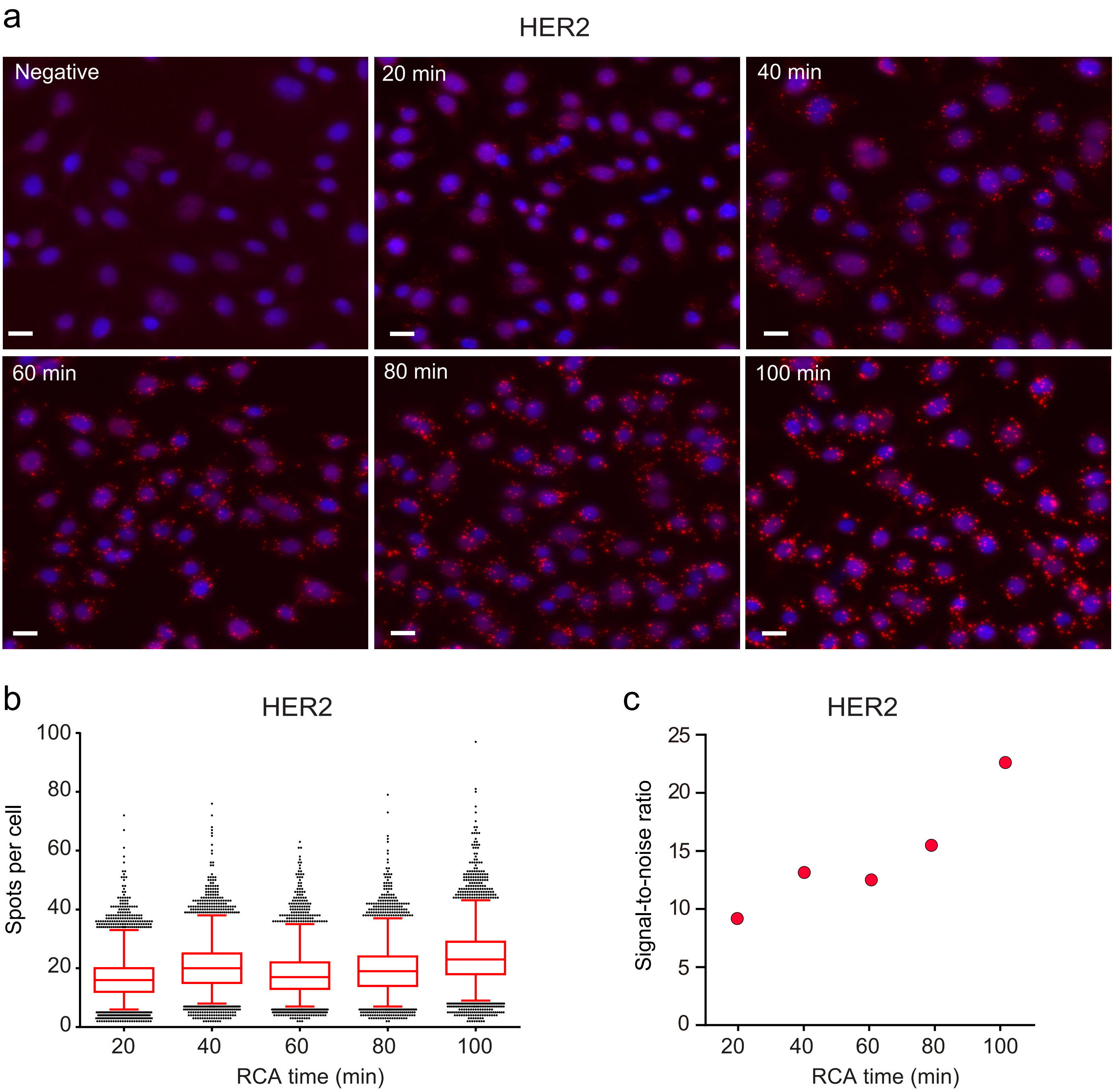

Supplementary Figure 1. Optimization of the RCA time in RollFISH. (a) Representative images of HER2 spots obtained with increasing RCA durations in A549 cells. Negative corresponds to a sample in which no RCA was performed. Blue, Nuclei. Scale bar, 10 µm. The experiment was repeated three times. **(b)** Quantification of HER2 spots in the images of which those in (a) are representative. Boxes extend from the 25th to the 75th percentile. Whiskers extend from 2.5 to 97.5 percentiles. The line inside each box represents the median value. (c) Signal-to-noise ratio of the images quantified in (b).

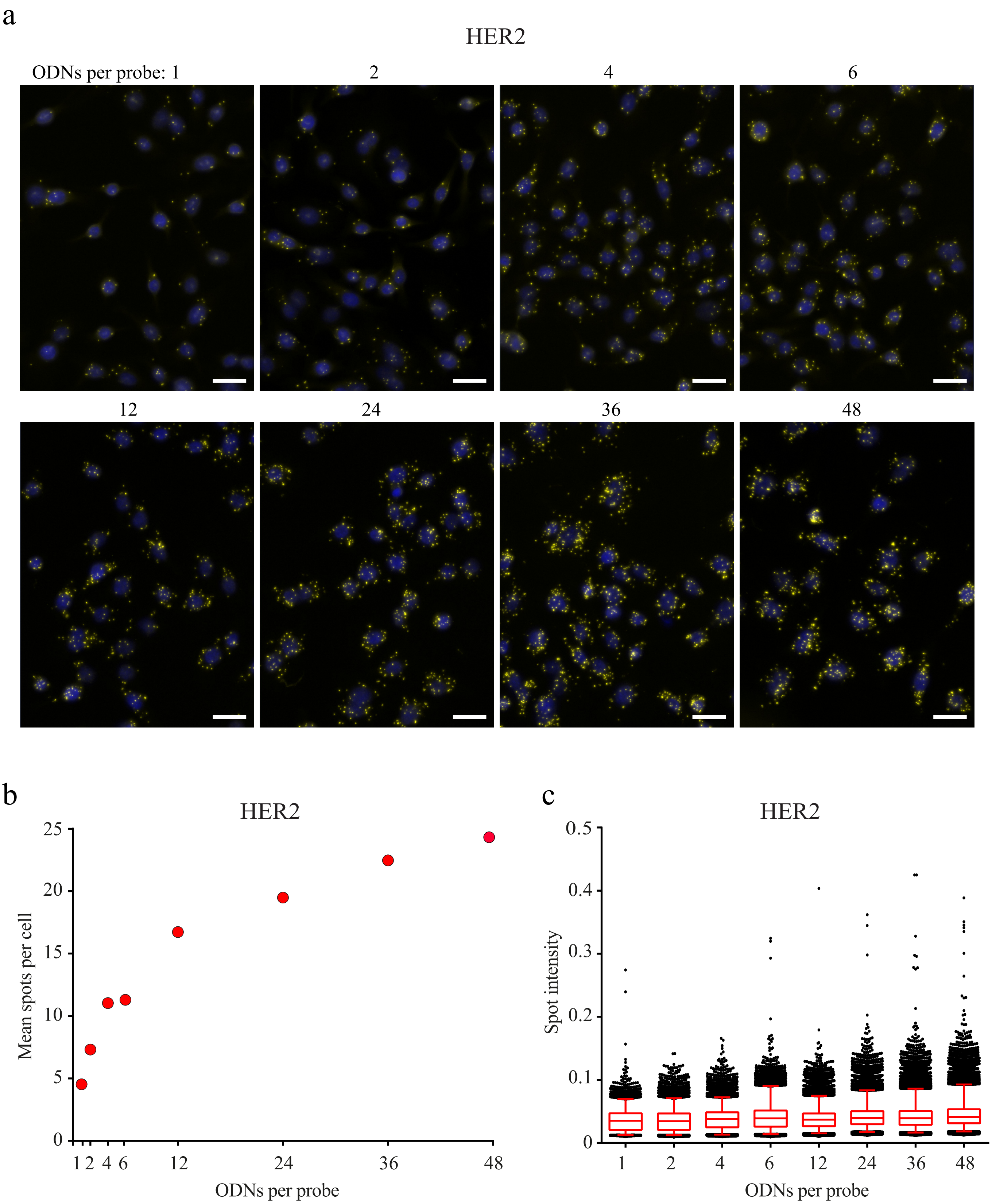

Supplementary Figure 2. Optimization of the number of ODNs per probe. (a) Representative images of HER2 spots obtained with an increasing number of ODNs per probe in A549 cells. Blue, Nuclei. Scale bar, 20 µm. **(b)** Quantification of HER2 spots in the images of which those in (a) are representative. The experiment was repeated three times, and the mean of three experiments is shown. **(c)** Intensity of HER2 spots in the images quantified in (b). Boxes extend from the 25th to the 75th percentile. Whiskers extend from 2.5 to 97.5 percentiles. The line inside each box represents the median value.

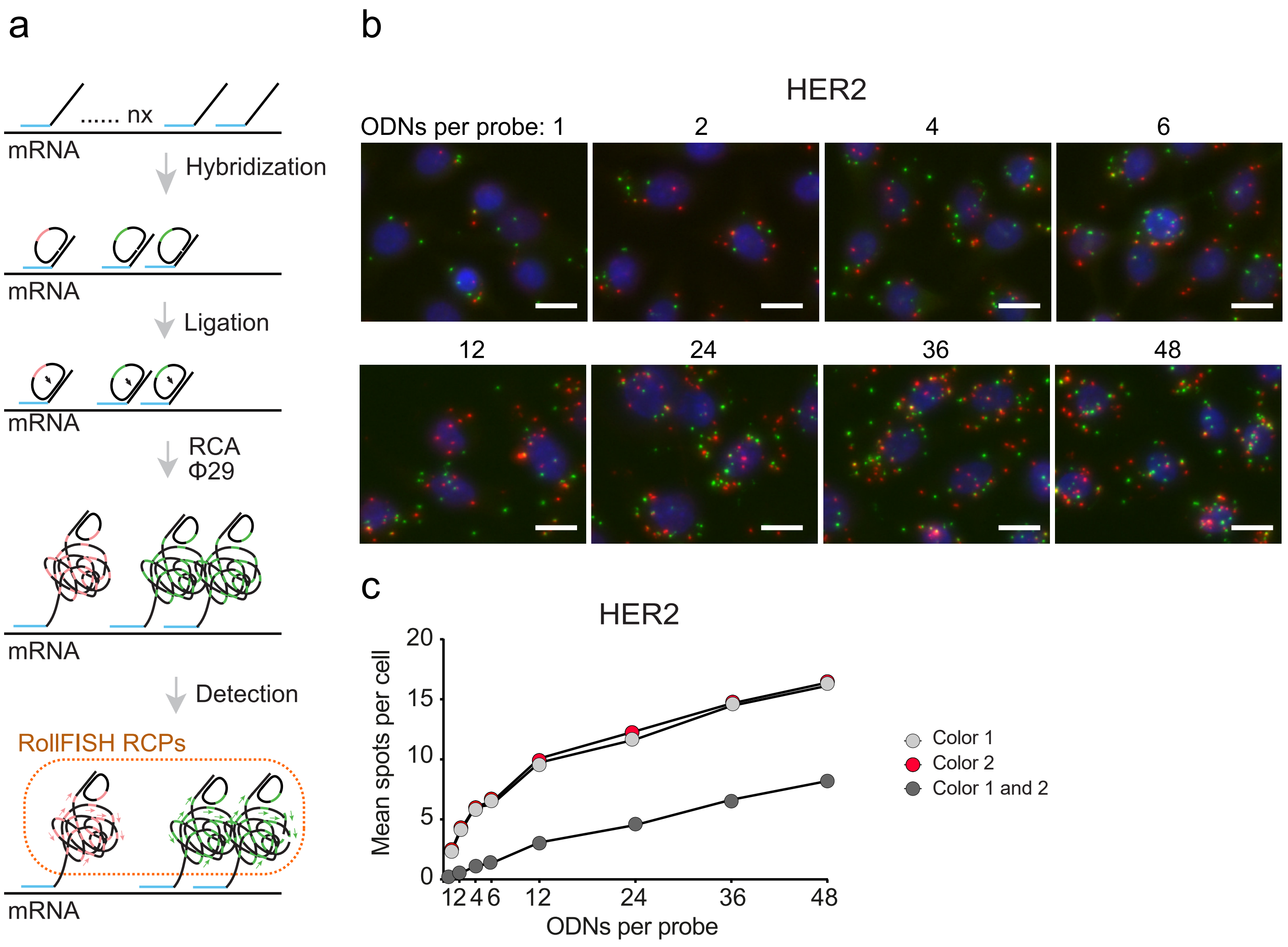

Supplementary Figure 3. Determination of RollFISH detection efficiency. (a) Schematic diagram of the colocalization assay. Two padlock probes were added at the same concentration to the same RCA reaction, thus competing for the same RollFISH probe ODNs. **(b)** Representative images of HER2 spots in two different colors, obtained with an increasing number of ODNs per probe in A549 cells. Blue, Nuclei. Scale bars, 10 µm. **(c)** Quantification of HER2 spots in the images of which those in (b) are representative. The experiment was repeated twice.

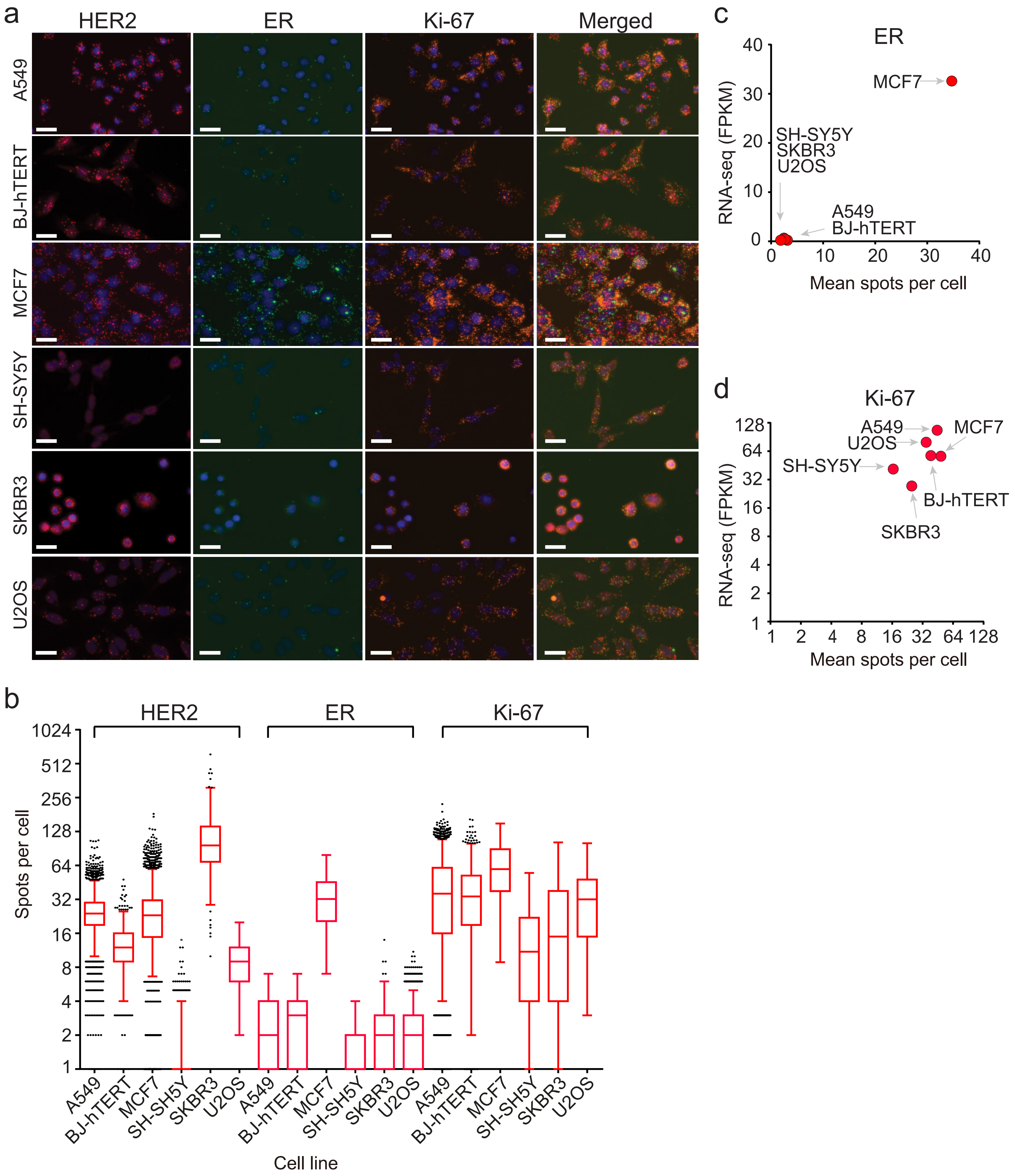

Supplementary Figure 4. Multiplex transcript detection in different cell lines. (a) Representative images of HER2, ER, and Ki-67 spots in six different cell lines. Probes consisted of 48 (HER2 and Ki-67) and 27 (ER) ODNs. Blue, Nuclei. Scale bar, 20 µm. **(b)** Quantification of the images of which those in (a) are representative. Boxes extend from the 25th to the 75th percentile. Whiskers extend from 2.5 to 97.5 percentiles. The line inside each box represents the median value. **(c)** Comparison of ER quantification by RollFISH and RNAseq in the six cell lines shown in (a), Pearson's R^2=0.99. **(d)** Same as in (c), but for Ki-67, Pearson's R^2=0.21.

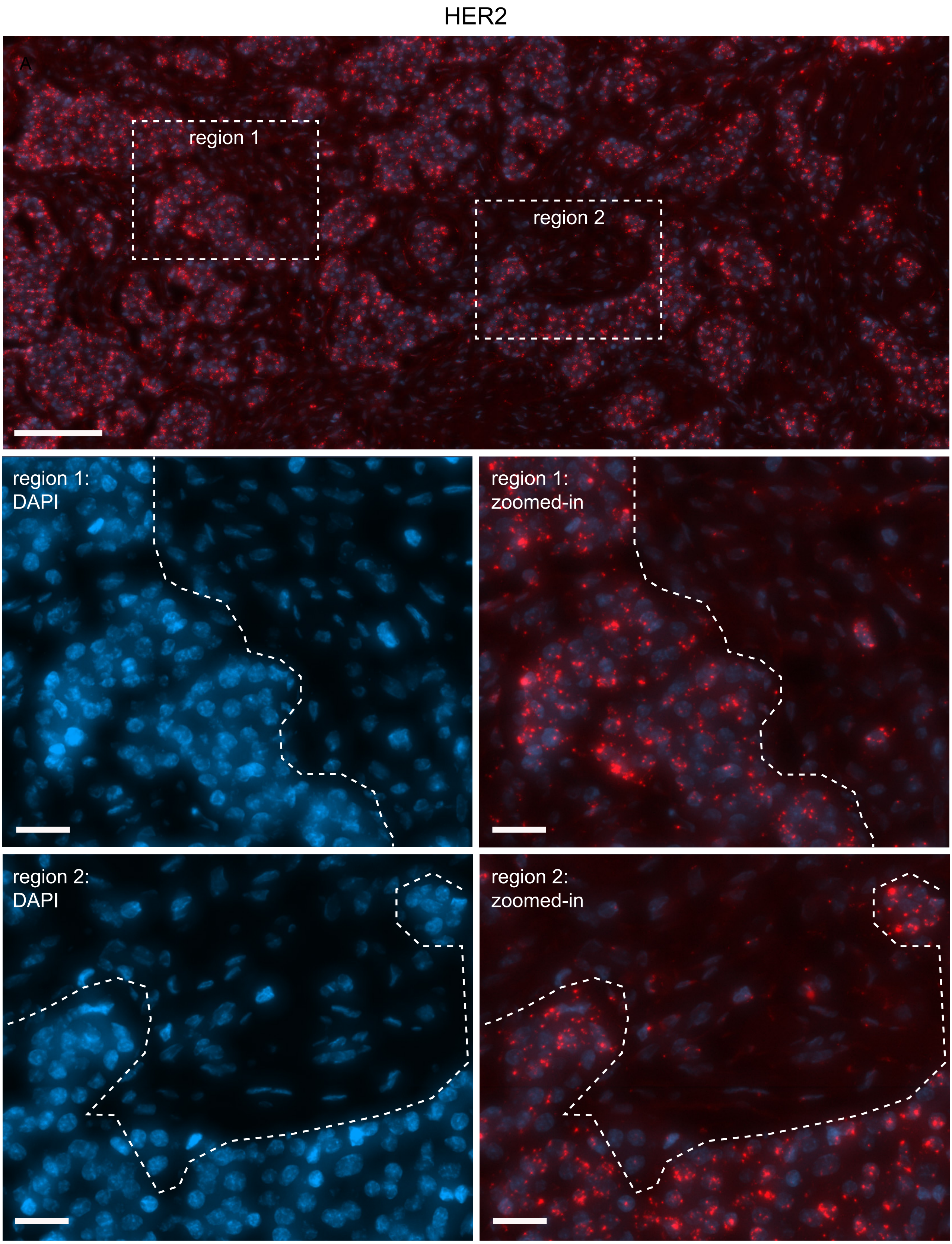

Supplementary Figure 5. HER2 detection in FFPE breast cancer tissues. Top image showing HER2 expression in a large tumor region. Blue, Nuclei. Scale bar, 100 µm. Two regions (region 1 and region 2) in dashed boxes were zoomed in and shown under the top image. Scale bar, 30 µm.

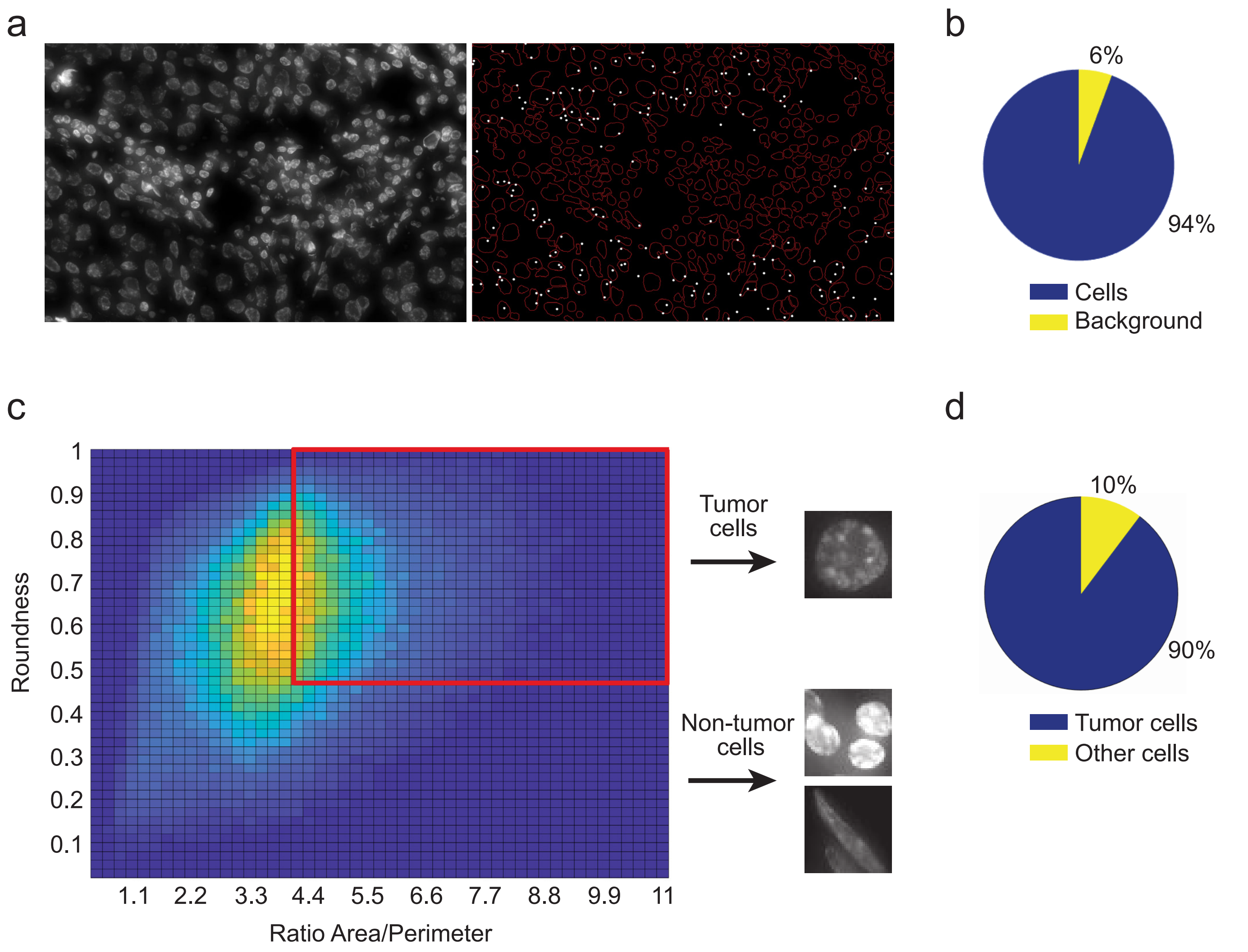

Supplementary Figure 6. Automatic cell segmentation and classification in FFPE tissues. (a) Representative image of DAPI-stained nuclei (left panel, grey) in tumor #8 (**Supplementary Table 2**), and HER2 spots (right panel, white) overlapping with segmented nuclei (right panel, red). **(b)** Pie chart representing the percentage of HER2 spots falling inside (blue) or outside (yellow) segmented cells in the same tumor shown in (a). **(c)** Heatmap scatterplot of area/perimeter ratio (x-axis) vs. roundness (y-axis) for all the segmented nuclei in the same tumor shown in (a). The red window shows putative tumor cells selected as described in **Methods**. Representative images of nuclei of tumor and non-tumor cells are shown on the right. **(d)** Pie chart showing the percentage of HER2 spots falling inside tumor cells vs. non-tumor cells defined as described in (c).

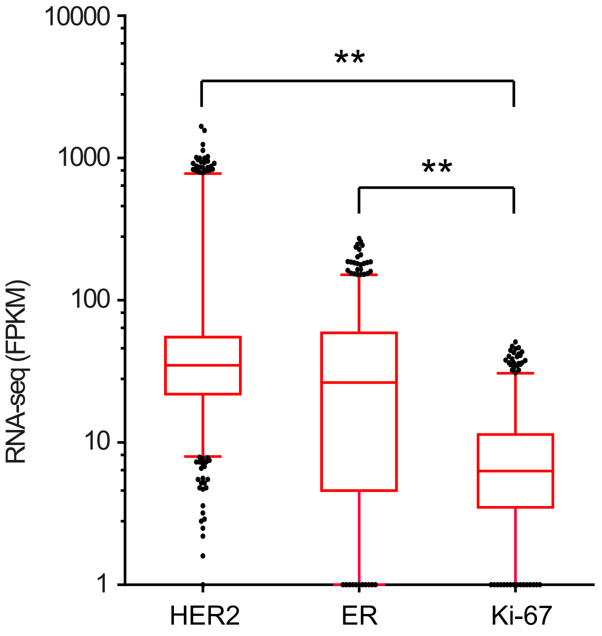

Supplementary Figure 7. HER2, ER, and Ki-67 RNA levels in TCGA (The Cancer Genome Atlas) breast cancers. RNA-seq data were downloaded from the Human Protein Atlas database (https://www.proteinatlas.org/). Boxplots are based on expression levels measured in 1,075 breast cancer samples. Boxes extend from the 25th to the 75th percentile. Whiskers extend from 2.5 to 97.5 percentiles. The line inside each box represents the median value.

HER2

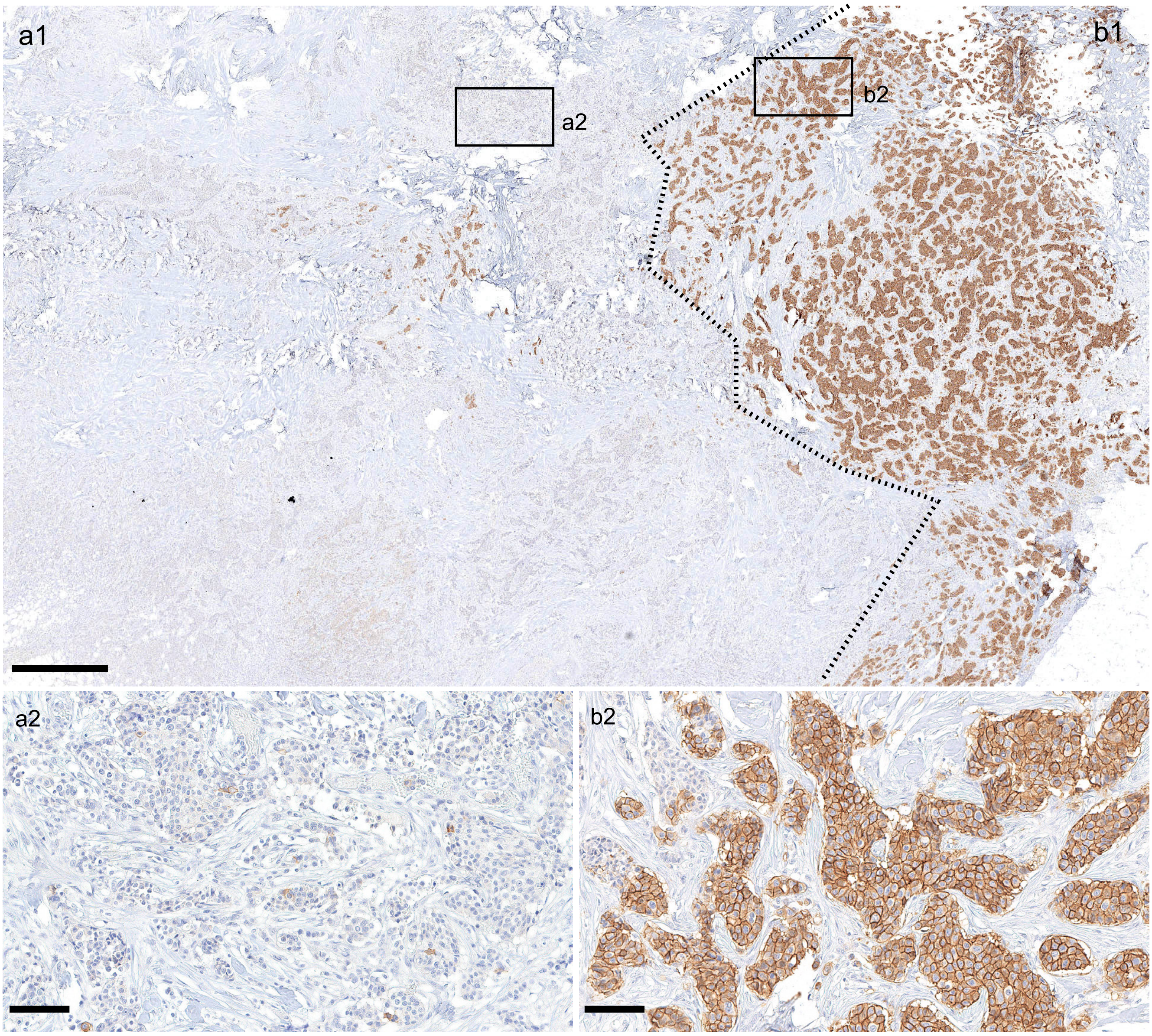

Supplementary Figure 8. Retrospective evaluation of HER2 by immunohistochemistry. Representative image from a retrospective evaluation of HER2 by IHC in tumor #8 (**Supplementary Table 2**). Two distinct tumor cell subpopulations, one HER2-negative (IHC 1+, 'a1') and the other HER2-positive (IHC 3+, 'b1'), were confirmed within the same tissue section. The black dashed line shows the border between these two populations. Scale bar, 1 mm. The areas marked as 'a2' and 'b2' are magnified below (scale bar, 100 μm).

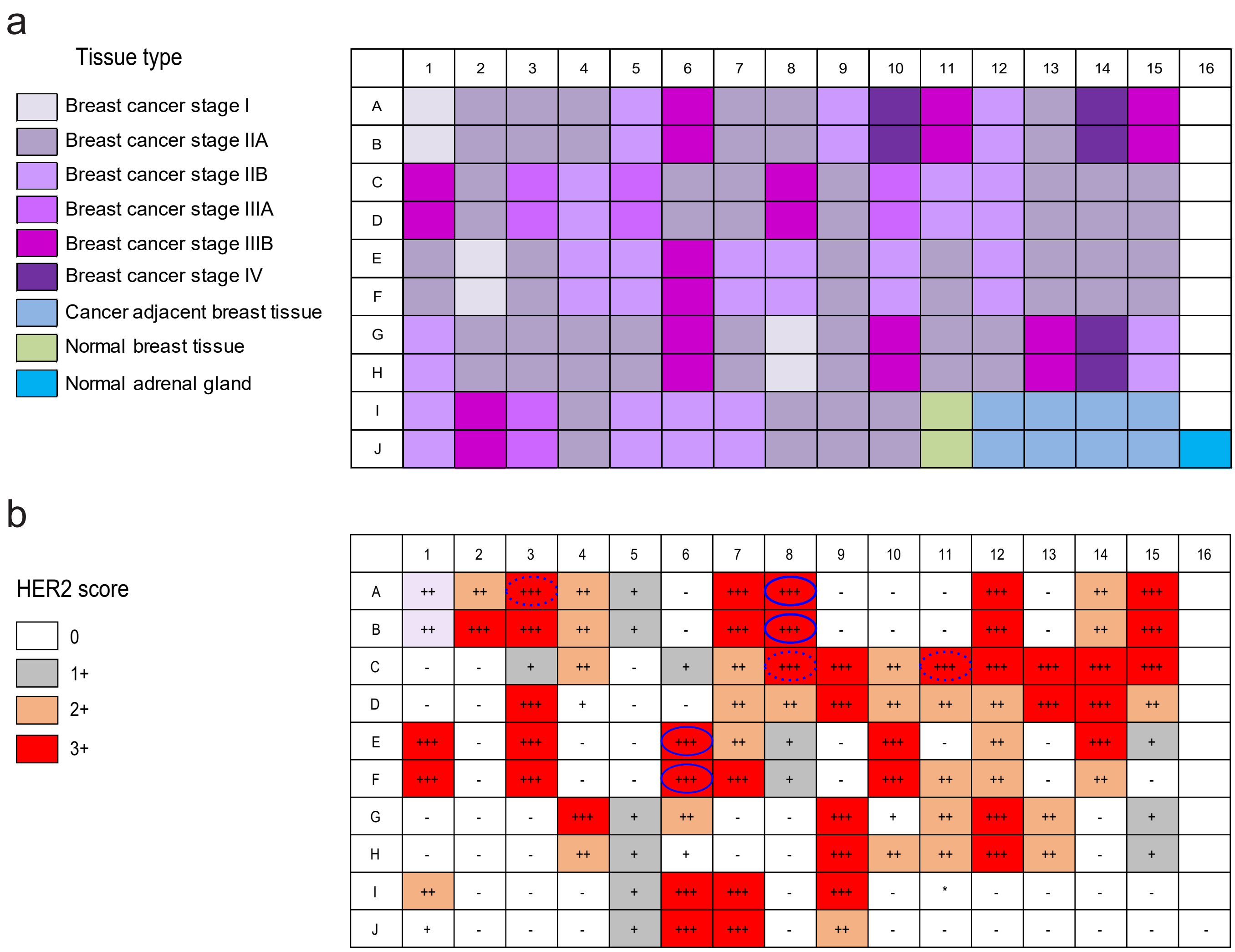

	1	2	3	4	5	6	7	8	9	10	11	12	13	14	15	16
A	++	++	+++	++	+	-	+++	+++	-	-	-	+++	-	++	+++	
B	++	+++	+++	++	+	-	+++	+++	-	-	-	+++	-	++	+++	
C	-	-	+	++	-	+	++	+++	+++	++	+++	+++	+++	+++	+++	
D	-	-	+++	+	-	-	++	++	+++	++	++	++	+++	+++	++	
E	+++	-	+++	-	-	+++	++	+	-	+++	-	++	-	+++	+	
F	+++	-	+++	-	-	+++	+++	+	-	+++	++	++	-	++	-	
G	-	-	-	+++	+	++	-	-	+++	+	++	+++	++	-	+	
H	-	-	-	++	+	+	-	-	+++	++	++	+++	++	-	+	
I	++	-	-	-	+	+++	+++	-	+++	-	*	-	-	-	-	
J	+	-	-	-	+	+++	+++	-	++	-	-	-	-	-	-	-

Supplementary Figure 9. Overview of the TMA used for HER2 RollFISH. (a) Position and tissue type of the 150 tissue cores on the TMA. An image of the TMA stained with hematoxylin-eosin is available at https://www.biomax.us/tissue-arrays/Breast/BR1504b. Each tissue is present in two duplicate cores (cores from the same tissue are found in the same column at the i-th and i-th+1 row). **(b)** Heatmap of the HER2 IHC score for all the samples shown in (a). HER2 score 3+ samples in which RollFISH detected a low HER2 transcript count were labeled with solid (<1 spot per cell) or dashed (1–1.5 spots per cell) blue circles.

Supplementary Table 1. List of ODNs and padlock probes used in this study.

Probe function	Probe ID	Sequence (5′–>3′)
Padlock probes	RCA-Ki-67	TTGCAAAATCATGGGCAGATAGTAGCCGTGACTATCGACTTGCGTCTATTTAGTGGAGCCGTACCTATCTTCTTTTTCGGAAGCAAATCTGATTG
	RCA-HER2	TGTGACTGTGTGGGAGCTGCCTCAATGCTGCTGCTGTACTACTGCGTCTATTTAGTGGAGCCAGCTCTATCTTCTTTAGAGTGATGTGTGGAGTTATGG
	RCA-ER	GAACCAGGGAAAATGTGTAGAGCGTGCGCCTGGTAGCAAATATGCGTCTATTTAGTGGAGCCCACGCTATCTTCTTTCTCCTAACTTGCTCTTGGACAG
	RollFISH-HER2_1	ACGCTTCGCTAACCCTACATGAGTCGTGAGTACGCTTTGCGTCTATTTAGTGGAGCCCGACCTATCTTCTTTACCGTAGTCGCTGCTATA
	RollFISH-HER2_2	ACGCTTCGCTAACCCTACCTCAATGCTGCTGCTGTACTACTGCGTCTATTTAGTGGAGCCCGACCTATCTTCTTTACCGTAGTCGCTGCTATA
	RollFISH-Ki-67_1	TACAGGGGTCAACATCGTACGTGCGCCTGGTAGCAAATATGCGTCTATTTAGTGGAGCCACGACTATCTTCTTTTACTACCCGCATCCCTAA
	RollFISH-Ki-67_2	TACAGGGGTCAACATCGTAGCGCCACTGATACTAGATTGTGCGTCTATTTAGTGGAGCCGACACTATCTTCTTTTACTACCCGCATCCCTAA
	RollFISH-ER_1	AGGTTGGACACGCAGATAAGTAGCCGTGACTATCGACTTGCGTCTATTTAGTGGAGCCGGCACTATCTTCTTTCGAGCTGAAGTGAATCCTA
	RollFISH-ER_2	AGGTTGGACACGCAGATACCTCAATGCACATGTTTGGCTCCTGCGTCTATTTAGTGGAGCCCCGACTATCTTCTTTCGAGCTGAAGTGAATCCTA
Detection probe	DO_Cy3	CAGTAAGGATAAGCAGCTGC
	DO_Cy3	AGTAGCCGTGACTATCGACT
	DO_TexasRed	CCTCAATGCTGCTGCTGTACTAC
	DO_Cy5	CATGAGACGTGAGTACGCTT
	DO_FITC	CGTGCGCCTGGTAGCAAATA
	DO_TexasRed	GCGCCACTGATACTAGATTG
Oligodeoxynucleotides (ODNs)		
HER2	HER2_2_ oligo	atatcctgcaggaaggacaggctggcattgTTTTCTAGCGTTAGGGTTAGCGAAGCGTTATAGCAGCGACTACGGT
	HER2_3_ oligo	tcacttggttgtgagcgatgagcacgtagcTTTTCTAGCGTTAGGGTTAGCGAAGCGTTATAGCAGCGACTACGGT
	HER2_4_ oligo	atagttgtcctcaaagagctgggtgcctcgTTTTCTAGCGTTAGGGTTAGCGAAGCGTTATAGCAGCGACTACGGT
	HER2_5_ oligo	gtattgttcagcgggtctccattgtctagcTTTTCTAGCGTTAGGGTTAGCGAAGCGTTATAGCAGCGACTACGGT
	HER2_7_ oligo	tccacaaaatcgtgtcctggtagcagagctTTTTCTAGCGTTAGGGTTAGCGAAGCGTTATAGCAGCGACTACGGT
	HER2_8_ oligo	tcagtgtgagagccagctggttgttcttgtTTTTCTAGCGTTAGGGTTAGCGAAGCGTTATAGCAGCGACTACGGT
	HER2_9_ oligo	tcaggctctgacaatcctcagaactctctcTTTTCTAGCGTTAGGGTTAGCGAAGCGTTATAGCAGCGACTACGGT
	HER2_11_ oligo	tcaaacgtgtctgtgttgtaggtgaccaggTTTTCTAGCGTTAGGGTTAGCGAAGCGTTATAGCAGCGACTACGGT
	HER2_12_ oligo	agtcacacagctggcgccgaatgtataccgTTTTCTAGCGTTAGGGTTAGCGAAGCGTTATAGCAGCGACTACGGT
	HER2_13_ oligo	aggatcccacgtccgtagaaaggtagttgtTTTTCTAGCGTTAGGGTTAGCGAAGCGTTATAGCAGCGACTACGGT
	HER2_14_ oligo	ttccatcctctgctgtcacctcttggttgtTTTTCTAGCGTTAGGGTTAGCGAAGCGTTATAGCAGCGACTACGGT
	HER2_15_ oligo	aagtgctccatgcccagaccatagcacactTTTTCTAGCGTTAGGGTTAGCGAAGCGTTATAGCAGCGACTACGGT
	HER2_16_ oligo	aactcctggatattggcactggtaactgccTTTTCTAGCGTTAGGGTTAGCGAAGCGTTATAGCAGCGACTACGGT
	HER2_17_ oligo	gcagaaatgccaggctcccaaagatcttctTTTTCTAGCGTTAGGGTTAGCGAAGCGTTATAGCAGCGACTACGGT
	HER2_18_ oligo	tccagagtctcaaacacttggagctgctctTTTTCTAGCGTTAGGGTTAGCGAAGCGTTATAGCAGCGACTACGGT
	HER2_19_ oligo	gtccggccatgctgagatgtataggtaaccTTTTCTAGCGTTAGGGTTAGCGAAGCGTTATAGCAGCGACTACGGT
	HER2_21_ oligo	gtcagcgagtaggcgccattgtgcagaattTTTTCTAGCGTTAGGGTTAGCGAAGCGTTATAGCAGCGACTACGGT
	HER2_22_ oligo	aagcagaggtgggtgttatggtggatgaggTTTTCTAGCGTTAGGGTTAGCGAAGCGTTATAGCAGCGACTACGGT
	HER2_23_ oligo	aaggaactggctgcagttgacacactgggtTTTTCTAGCGTTAGGGTTAGCGAAGCGTTATAGCAGCGACTACGGT
	HER2_24_ oligo	aaacagtgcctggcattcacatactccctgTTTTCTAGCGTTAGGGTTAGCGAAGCGTTATAGCAGCGACTACGGT
	HER2_25_ oligo	aaacaggtcactgagccattctggggctgaTTTTCTAGCGTTAGGGTTAGCGAAGCGTTATAGCAGCGACTACGGT
	HER2_26_ oligo	ttatagtgggcacaggccacacactggtcaTTTTCTAGCGTTAGGGTTAGCGAAGCGTTATAGCAGCGACTACGGT
	HER2_27_ oligo	atgtaggagaggtcaggtttcacaccgctgTTTTCTAGCGTTAGGGTTAGCGAAGCGTTATAGCAGCGACTACGGT
	HER2_28_ oligo	ctggcatgcgccctcctcatctggaaacttTTTTCTAGCGTTAGGGTTAGCGAAGCGTTATAGCAGCGACTACGGT
	HER2_29_ oligo	atccaggtccacacaggagtgggtgcagttTTTTCTAGCGTTAGGGTTAGCGAAGCGTTATAGCAGCGACTACGGT
	HER2_30_ oligo	aatgccaaccaccgcagagatgatggacgtTTTTCTAGCGTTAGGGTTAGCGAAGCGTTATAGCAGCGACTACGGT
	HER2_31_ oligo	tgatgaggatcccaaagaccacccccaagaTTTTCTAGCGTTAGGGTTAGCGAAGCGTTATAGCAGCGACTACGGT
	HER2_32_ oligo	tctccgcatcgtgtacttccggatcttctgTTTTCTAGCGTTAGGGTTAGCGAAGCGTTATAGCAGCGACTACGGT
	HER2_33_ oligo	tcagctccgtctctttcaggatccgcatctTTTTCTAGCGTTAGGGTTAGCGAAGCGTTATAGCAGCGACTACGGT
	HER2_34_ oligo	tgtagactgtgccaaaagcgccagatccaaTTTTCTAGCGTTAGGGTTAGCGAAGCGTTATAGCAGCGACTACGGT
	HER2_35_ oligo	ggaattttcacattctccccatcagggatcTTTTCTAGCGTTAGGGTTAGCGAAGCGTTATAGCAGCGACTACGGT
	HER2_36_ oligo	gctttgggggatgtgttttccctcaacactTTTTCTAGCGTTAGGGTTAGCGAAGCGTTATAGCAGCGACTACGGT
	HER2_38_ oligo	aggcagatgcccagaaggcgggagacatatTTTTCTAGCGTTAGGGTTAGCGAAGCGTTATAGCAGCGACTACGGT
	HER2_39_ oligo	taagaggcagccatagggcataagctgtgtTTTTCTAGCGTTAGGGTTAGCGAAGCGTTATAGCAGCGACTACGGT
	HER2_41_ oligo	tacgagccgcacatcctccaggtagctcatTTTTCTAGCGTTAGGGTTAGCGAAGCGTTATAGCAGCGACTACGGT
	HER2_42_ oligo	tttgacatggttgggactcttgaccagcacTTTTCTAGCGTTAGGGTTAGCGAAGCGTTATAGCAGCGACTACGGT
	HER2_43_ oligo	atggtactctgtctcgtcaatgtccagcagTTTTCTAGCGTTAGGGTTAGCGAAGCGTTATAGCAGCGACTACGGT
	HER2_46_ oligo	taaggtttggccccaaaagtcatcagctccTTTTCTAGCGTTAGGGTTAGCGAAGCGTTATAGCAGCGACTACGGT
	HER2_48_ oligo	ggccgacattcagagtcaatcatccaacatTTTTCTAGCGTTAGGGTTAGCGAAGCGTTATAGCAGCGACTACGGT
	HER2_49_ oligo	tggccatgcgggagaattcagacaccaactTTTTCTAGCGTTAGGGTTAGCGAAGCGTTATAGCAGCGACTACGGT
	HER2_51_ oligo	tgagcggtagaaggtgctgtccaagggactTTTTCTAGCGTTAGGGTTAGCGAAGCGTTATAGCAGCGACTACGGT
	HER2_53_ oligo	ttcccaggtcaccatcaaatacatcggagcTTTTCTAGCGTTAGGGTTAGCGAAGCGTTATAGCAGCGACTACGGT
	HER2_54_ oligo	actgtggggtcctcactgtaccgctgtagaTTTTCTAGCGTTAGGGTTAGCGAAGCGTTATAGCAGCGACTACGGT
	HER2_55_ oligo	caggggggcaacgtagccatcagtctcagaTTTTCTAGCGTTAGGGTTAGCGAAGCGTTATAGCAGCGACTACGGT
	HER2_56_ oligo	acatctggctggttcacatattcaggctggTTTTCTAGCGTTAGGGTTAGCGAAGCGTTATAGCAGCGACTACGGT

	HER2_57_ oligo	agagtcttgggcctttccagagtggcaccaTTTTCTAGCGTTAGGGTTAGCGAAGCGTTATAGCAGCGACTACGGT
	HER2_58_ oligo	ccaaaggcaaaaacgtctttgacgaccccaTTTTCTAGCGTTAGGGTTAGCGAAGCGTTATAGCAGCGACTACGGT
	HER2_59_ oligo	taatagaggttgtcgaaggctgggctgaagTTTTCTAGCGTTAGGGTTAGCGAAGCGTTATAGCAGCGACTACGGT
ER	ER_1_ oligo	cagatgctttggtgtggagggtcatggtcaTTTTATGTATATCTGCGTGTCCAACCTTAGGATTCACTTCAGCTCG
	ER_2_ oligo	gctccagctcgttcccttggatctgatgcaTTTTATGTATATCTGCGTGTCCAACCTTAGGATTCACTTCAGCTCG
	ER_3_ oligo	agttgtacacggcgggcttgctgctgtccaTTTTATGTATATCTGCGTGTCCAACCTTAGGATTCACTTCAGCTCG
	ER_4_ oligo	tcggagacacgctgttgagtggggggaaacTTTTATGTATATCTGCGTGTCCAACCTTAGGATTCACTTCAGCTCG
	ER_5_ oligo	tcgttctccaggtagtagggcacctgctggTTTTATGTATATCTGCGTGTCCAACCTTAGGATTCACTTCAGCTCG
	ER_7_ oligo	ttccatagccatacttcccttgtcattggTTTTTATGTATATCTGCGTGTCCAACCTTAGGATTCACTTCAGCTCG
	ER_8_ oligo	cattgcacactgcacagtagcgagtctccTTTTTATGTATATCTGCGTGTCCAACCTTAGGATTCACTTCAGCTCG
	ER_9_ oligo	ggaccagactccataatggtagcctgaagcTTTTATGTATATCTGCGTGTCCAACCTTAGGATTCACTTCAGCTCG
	ER_10_ oligo	tggtggctggacacatatagtcgttatgtcTTTTATGTATATCTGCGTGTCCAACCTTAGGATTCACTTCAGCTCG
	ER_11_ oligo	cttcctcctgttttatcaatggtgcactgTTTTATGTATATCTGCGTGTCCAACCTTAGGATTCACTTCAGCTCG
	ER_12_ oligo	tcttttcgtatcccaccttttcatcattccTTTTATGTATATCTGCGTGTCCAACCTTAGGATTCACTTCAGCTCG
	ER_13_ oligo	ggcgcttgtgtttcaacattctccctcctcTTTTATGTATATCTGCGTGTCCAACCTTAGGATTCACTTCAGCTCG
	ER_14_ oligo	atgagcgggcttggccaaaggttggcagcTTTTTATGTATATCTGCGTGTCCAACCTTAGGATTCACTTCAGCTCG
	ER_15_ oligo	acaaggccaggctgttcttcttagagcgTTTTTTATGTATATCTGCGTGTCCAACCTTAGGATTCACTTCAGCTCG
	ER_16_ oligo	agggtctggtaggatcatactcggaatagaTTTTATGTATATCTGCGTGTCCAACCTTAGGATTCACTTCAGCTCG
	ER_17_ oligo	tggtcagtaagcccatcatcgaagcttcacTTTTATGTATATCTGCGTGTCCAACCTTAGGATTCACTTCAGCTCG
	ER_18_ oligo	cagttgatcatgtgaaccagctccctgtcTTTTTATGTATATCTGCGTGTCCAACCTTAGGATTCACTTCAGCTCG
	ER_19_ oligo	attctagaaggtggacctgatcatggagggTTTTATGTATATCTGCGTGTCCAACCTTAGGATTCACTTCAGCTCG
	ER_20_ oligo	gacgagaccaatcatcaggatctcagccaTTTTATGTATATCTGCGTGTCCAACCTTAGGATTCACTTCAGCTCG
	ER_21_ oligo	tttccctggttcctgtccaagagcaagttaTTTTATGTATATCTGCGTGTCCAACCTTAGGATTCACTTCAGCTCG
	ER_22_ oligo	catgtcgaagatctccaccatgccctctacTTTTATGTATATCTGCGTGTCCAACCTTAGGATTCACTTCAGCTCG
	ER_23_ oligo	ttcatcatgcggaaccgagatgatgtagccTTTTATGTATATCTGCGTGTCCAACCTTAGGATTCACTTCAGCTCG
	ER_25_ oligo	atatggtccttctcttccagagacttcaggTTTTATGTATATCTGCGTGTCCAACCTTAGGATTCACTTCAGCTCG
	ER_26_ oligo	aaagtgctgtgatcttgtccaggactcggTTTTATGTATATCTGCGTGTCCAACCTTAGGATTCACTTCAGCTCG
	ER_28_ oligo	cagatgctccatgcctttgttactcatgtgTTTTATGTATATCTGCGTGTCCAACCTTAGGATTCACTTCAGCTCG
	ER_29_ oligo	catagagggcaccacgttcttgcacttcaTTTTATGTATATCTGCGTGTCCAACCTTAGGATTCACTTCAGCTCG
	ER_30_ oligo	agtggctttggtccgtctcctccacggatgTTTTATGTATATCTGCGTGTCCAACCTTAGGATTCACTTCAGCTCG
Ki-67	Ki-67_ 2_ oligo	aatacccccttccaaacaagcaggtgctgagTTTTAGTGTTACGATGTTGACCCCTGTATTAGGGATGCGGGTAGTA
	Ki-67_ 4_ oligo	gacgtgctggctcctgttcacgtatttttcTTTTAGTGTTACGATGTTGACCCCTGTATTAGGGATGCGGGTAGTA
	Ki-67_ 6_ oligo	gagcaacactgtcttttgagtcatctgcggTTTTAGTGTTACGATGTTGACCCCTGTATTAGGGATGCGGGTAGTA
	Ki-67_ 7_ oligo	gccattacgtccagcatgttctgaggaatgTTTTAGTGTTACGATGTTGACCCCTGTATTAGGGATGCGGGTAGTA
	Ki-67_ 10_ oligo	tcaagctcttgttcaggtgaagcaggctctTTTTAGTGTTACGATGTTGACCCCTGTATTAGGGATGCGGGTAGTA
	Ki-67 _11_ oligo	agtctgaacagactccacgtctcttcccttTTTTAGTGTTACGATGTTGACCCCTGTATTAGGGATGCGGGTAGTA
	Ki-67 _12_ oligo	ttttagccggctcatagagaggaaagctggTTTTAGTGTTACGATGTTGACCCCTGTATTAGGGATGCGGGTAGTA
	Ki-67 _13_ oligo	ccagccttgaagccttcacttttacccagaTTTTAGTGTTACGATGTTGACCCCTGTATTAGGGATGCGGGTAGTA
	Ki-67_14_ oligo	ggcagagtcagctgcatcttcaactttagcTTTTAGTGTTACGATGTTGACCCCTGTATTAGGGATGCGGGTAGTA
	Ki-67_15_ oligo	ccgtaggcagaacttccacatctgtaggaaTTTTAGTGTTACGATGTTGACCCCTGTATTAGGGATGCGGGTAGTA
	Ki-67_16_ oligo	cttcctctcaacttgagtgagccacagagtTTTTAGTGTTACGATGTTGACCCCTGTATTAGGGATGCGGGTAGTA
	Ki-67 _17_ oligo	tgcccaatttctcaggcttgctgagggaatTTTTAGTGTTACGATGTTGACCCCTGTATTAGGGATGCGGGTAGTA
	Ki-67_18_ oligo	ctaagaccaggtaacccagagcacatctgtTTTTAGTGTTACGATGTTGACCCCTGTATTAGGGATGCGGGTAGTA
	Ki-67_19_ oligo	cccaccaaaggacacacgccttcttttcaaTTTTAGTGTTACGATGTTGACCCCTGTATTAGGGATGCGGGTAGTA
	Ki-67_20_ oligo	ccctttttgagaggcgtattaggaggcaagtTTTTAGTGTTACGATGTTGACCCCTGTATTAGGGATGCGGGTAGTA
	Ki-67_21_ oligo	caggtggagtgtgcattaccagagactttcTTTTAGTGTTACGATGTTGACCCCTGTATTAGGGATGCGGGTAGTA
	Ki-67_23_ oligo	ggagggcttataaccaagctttgtgccttcTTTTAGTGTTACGATGTTGACCCCTGTATTAGGGATGCGGGTAGTA
	Ki-67_24_ oligo	gcgttgatcactggcaactggagttttcctTTTTAGTGTTACGATGTTGACCCCTGTATTAGGGATGCGGGTAGTA
	Ki-67_26_ oligo	tgccactctttctccctcctctcttaggaaTTTTAGTGTTACGATGTTGACCCCTGTATTAGGGATGCGGGTAGTA
	Ki-67_27_ oligo	gcttctttggagtagcaggtcttctttgccTTTTAGTGTTACGATGTTGACCCCTGTATTAGGGATGCGGGTAGTA
	Ki-67_28_ oligo	gtacaaggagagtttgcgtggcctgtactaTTTTAGTGTTACGATGTTGACCCCTGTATTAGGGATGCGGGTAGTA
	Ki-67_29_ oligo	gggtcgagcaggcacatgtactttttcagtTTTTAGTGTTACGATGTTGACCCCTGTATTAGGGATGCGGGTAGTA
	Ki-67_30_ oligo	ctccttcactggggtcttgaacatttcagcTTTTAGTGTTACGATGTTGACCCCTGTATTAGGGATGCGGGTAGTA
	Ki-67_32_ oligo	ggctgttttgctgcattctgtgcactgaagTTTTAGTGTTACGATGTTGACCCCTGTATTAGGGATGCGGGTAGTA
	Ki-67_33_ oligo	taatacactgccgtcttaagggagggcttgTTTTAGTGTTACGATGTTGACCCCTGTATTAGGGATGCGGGTAGTA
	Ki-67_34_ oligo	gtaggtgttcctgggcgtttttgctacgttTTTTAGTGTTACGATGTTGACCCCTGTATTAGGGATGCGGGTAGTA
	Ki-67_35_ oligo	ccttcctgacctgtttgcagtggatactgtTTTTAGTGTTACGATGTTGACCCCTGTATTAGGGATGCGGGTAGTA
	Ki-67_36_ oligo	ctgcccccaagttcttgatctcttcattgcTTTTAGTGTTACGATGTTGACCCCTGTATTAGGGATGCGGGTAGTA
	Ki-67_37_ oligo	agctcttgaggtctgtcaggtcagacattgTTTTAGTGTTACGATGTTGACCCCTGTATTAGGGATGCGGGTAGTA
	Ki-67_39_ oligo	ggttctggttgtaatgactggcagggcattTTTTAGTGTTACGATGTTGACCCCTGTATTAGGGATGCGGGTAGTA
	Ki-67_40_ oligo	cccagggatgccttcaactgttgttttgtgTTTTAGTGTTACGATGTTGACCCCTGTATTAGGGATGCGGGTAGTA
	Ki-67_41_ oligo	acttgccgactgctaggagctcttctttcaTTTTAGTGTTACGATGTTGACCCCTGTATTAGGGATGCGGGTAGTA
	Ki-67_42_ oligo	aacgttctgatgctcttgccatctcctgctTTTTAGTGTTACGATGTTGACCCCTGTATTAGGGATGCGGGTAGTA
	Ki-67_43_ oligo	acttcttcattccagttacacgggctgctgTTTTAGTGTTACGATGTTGACCCCTGTATTAGGGATGCGGGTAGTA

Ki-67_45_ oligo	tcattgattcctcagagggacctggtgtctTTTTAGTGTTACGATGTTGACCCCTGTATTAGGGATGCGGGTAGTA
Ki-67_46_ oligo	ctggtggtggagatttgcaggctattttggTTTTAGTGTTACGATGTTGACCCCTGTATTAGGGATGCGGGTAGTA
Ki-67_47_ oligo	gctttcctgagacttctcttaggccattgcTTTTAGTGTTACGATGTTGACCCCTGTATTAGGGATGCGGGTAGTA
Ki-67_48_ oligo	cctgctgatggtgttagtttcctgagtgctTTTTAGTGTTACGATGTTGACCCCTGTATTAGGGATGCGGGTAGTA
Ki-67_49_ oligo	tgtctttctcatcacctcctgctggtttggTTTTAGTGTTACGATGTTGACCCCTGTATTAGGGATGCGGGTAGTA
Ki-67_50_ oligo	aggtccagtttctgcactggagttcccataTTTTAGTGTTACGATGTTGACCCCTGTATTAGGGATGCGGGTAGTA
Ki-67_51_ oligo	gagtctgtagctgtcttttgctgccaggtaTTTTAGTGTTACGATGTTGACCCCTGTATTAGGGATGCGGGTAGTA
Ki-67_53_ oligo	ttaccagcagccactaattcctcggtgtgaTTTTAGTGTTACGATGTTGACCCCTGTATTAGGGATGCGGGTAGTA
Ki-67_54_ oligo	tctcttgggtcgttgctttgtgcttgttggTTTTAGTGTTACGATGTTGACCCCTGTATTAGGGATGCGGGTAGTA
Ki-67_55_ oligo	cacgctaagagttctccctctacatctgctTTTTAGTGTTACGATGTTGACCCCTGTATTAGGGATGCGGGTAGTA
Ki-67_56_ oligo	actgatggtttaggcgtgtgcatggctttgTTTTAGTGTTACGATGTTGACCCCTGTATTAGGGATGCGGGTAGTA
Ki-67_57_ oligo	gtccagtttctgcactggagttcccacaaaTTTTAGTGTTACGATGTTGACCCCTGTATTAGGGATGCGGGTAGTA
Ki-67_58_ oligo	ttccttaggagtttgtggccgtctcttgctTTTTAGTGTTACGATGTTGACCCCTGTATTAGGGATGCGGGTAGTA

Sequences with underline are backbone sequence designed in padlock probes; Sequences in lower case letters in primary oligonucleotides are complementary sequences to mRNA targets.

Supplementary Table 2. Pathological information on breast cancer samples included in this study.

Tumor ID	Age	Sex	Cancer Molecular Subtype	HER2 (IHC score)	ER, %	PR, %	Ki-67, %
1	42	F	Triple negative	0	0	0	70
2	47	F	Triple negative	0	0	0	90
3	54	F	HER2+	3	0	0	55
4	57	F	HER2+	3	0	0	5
5	79	F	Luminal A	0	99	95	5
6	81	F	Luminal B (HER2-)	0	99	65	32
7	67	F	Luminal B (HER2-)	0	90	2	70
8	57	F	Luminal B (HER2+)	3	99	70	45
9	68	F	Luminal B (HER2+)	3	95	60	13

Supplementary Table 3. Comparison of HER2, ER and Ki-67 quantification by RollFISH vs. IHC.

Sample ID	Pathological information	Dots per cell	% Cells in Bin 0, <1 dot/cell	% Cells in Bin 1, 1 dots/cell	% Cells in Bin 2, 2-4 dots/cel	% Cells in Bin 3, 5-9 dots/cel	% Cells in Bin 4, >10 dots/cel	H-score	% Positive Cells
Tissue No.1	Subtype: Triple negative								
	HER2 IHC score: 0	0,00168	99,9	0,07	0,012	0,0039	0,0039	0,13	0,09
	ER1: 0%	0,0308	97,2	0,027	0,014	0,012	0,0039	0,096	2,8
	Ki-67: 70%	0,0473	95,9	3,684	0,39	0,012	0,0078	4,52	4,1
Tissue No.2	Subtype: Triple negative								
	HER2 IHC score: 0	0,0822	94	4,8	1,1	0,1	0,017	7,37	6
	ER1: 0%	0,0814	94,1	4,5	1,3	0,068	0,015	7,36	5,9
	Ki-67: 90%	0,133	92,3	5,5	1,8	0,35	0,077	10,5	7,7
Tissue No.3	Subtype: HER2+								
	HER2 IHC score: 3+	5,735	16,7	10,88	23,1	27,5	21,85	238,1	83,3
	ER1: 0%	0,0617	94,6	4,8	0,55	0,018	0	6	5,4
	Ki-67: 55%	0,158	87,8	9,8	2,26	0,12	0,014	14,7	12,2
Tissue No.4	Subtype: HER2+								
	HER2 IHC score: 3+	0,561	68,6	15,7	12,5	2,93	0,25	40,5	31,4
	ER1: 0%	0,122	91,9	5,7	0,02	0,0017	0,00053	11,5	8
	Ki-67: 5%	0,0933	94,6	4,1	1	0,03	0,00053	6,2	5,4
Tissue No.5	Subtype: Luminal A								
	HER2 (IHC score): 0	0,0201	98,4	1,48	0,13	0,017	0,0087	1,83	1,65
	ER1: 99%	0,795	55,1	25,35	18	1,46	0,044	65,9	44,9
	Ki-67: 5%	0,126	90,8	7,51	1,45	0,14	0,053	11,1	9,2
Tissue No.6	Subtype: Luminal B (HER2-)								
	HER2 IHC score: 0	0,0939	98,39	6,26	0,51	0,0105	0	7,31	1,6
	ER1: 99%	1,587	26,88	26,87	34,89	6,35	0,17	116,38	73,13
	Ki-67: 32%	0,0889	92,76	6,33	0,83	0,067	0,022	8,28	7,24
Tissue No.7	Subtype: Luminal B (HER2-)								
	HER2 IHC score: 0	0,0814	93,7	5,1	1,11	0,069	0,0040	7,5	6,3
	ER1: 90%	0,543	71,5	15,1	12	1,38	0,055	43,46	28,5
	Ki-67: 70%	0,0429	77,1	14,6	7,9	0,39	0,0094	4,03	3,6
Tissue No.8	Subtype: Luminal B (HER2+)								
	HER2 IHC score: 3+	0,257	82,1	12,8	4,84	0,24	0,018	23,27	17,9
	ER1: 99%	1,553	47,5	19,1	23,3	8,5	1,58	97,42	52,5
	Ki-67: 45%	0,233	84,8	10,7	4,1	0,35	0,057	20	15,2
Tissue No.9	Subtype: Luminal B (HER2+)								
	HER2 IHC score: 3+	0,667	65,2	18,6	14,4	1,7	0,17	53,2	34,8
	ER1: 95%	2,141	37,4	17,1	29,5	13,7	2,3	126,4	62,6
	Ki67: 13%	0,0987	92	6,6	1,4	0,024	0,0018	9,5	8,02

During analysis, cells on tissues were classified into five bins according to the expression level of each gene in individual cells, and 0-4 score were assigned to each cell in these five bins accordingly.

Supplementary Table 4. Pathological information on tumors in the TMA.

**Age at diagnosis (year old)**	**Number of cores (%)**	**Number of cases (%)**
<40		18 (24%)
40-50		26 (34.7%)
51-70		30 (40%)
>70		1 (1.3%)
		75
**Pathology grade**	**Number of cores (%)**	**Number of cases (%)**
1	33 (22%)	16.5 (22%)
2	93 (62%)	46 (61.3%)
3	10 (6.7%)	5.5 (7.3%)
Undetermined	14 (9.3%)	7 (9.3%)
Total	150	75
**HER2 status (IHC score)**		
0	61 (40.7%)	30.5 (40.7%)
1+	17 (11.3%)	8.5 (11.3%)
2+	30 (20%)	15 (20%)
3+	42 (28%)	21 (28%)
Total	150	75
**Progesterone receptor (PR) score**		
> 5% (range from 1+ to 3+)	48 (32%)	24 (32%)
Negative	102 (68%)	51 (68%)
Total	150	75
**ER status**		
> 5% (range from 1+ to 3+)	44 (29.3%)	22 (29.3%)
Negative	106 (70.7%)	53 (70.7%)
Total	150	75
**Disease Stage**		
Negative	10 (6.7%)	5 (6.7%)
Stage I	6 (4%)	3 (4%)
Stage IIA	64 (42.7%)	32 (42.7%)
Stage IIB	36 (24%)	18 (24%)
Stage IIIA	8 (5.3%)	4 (5.3%)
Stage IIIB	20 (13.3%)	10 (13.3%)
Stage IV	6 (4%)	3 (4%)
Total	150	75

Supplementary Table 5. Pathological and clinical information on tumors in the TMA

Cases	75
Cores	150
Core diameter	1 mm
Rows	10
Thickness	5 µm

No.	Position	Age	Sex	Organ/Anatomic Site	Pathology diagnosis	Grade	Stage	Type	ER	PR	HER2
1	A1	46	F	Breast	Invasive ductal carcinoma	1	I	Malignant	-	++, 40%	++
2	A2	36	F	Breast	Invasive ductal carcinoma	1	IIA	Malignant	-	-	++
3	A3	31	F	Breast	Invasive ductal carcinoma	1	IIA	Malignant	-	-	+++
4	A4	60	F	Breast	Invasive ductal carcinoma	1	IIA	Malignant	-	-	++
5	A5	53	F	Breast	Invasive ductal carcinoma	1	IIB	Malignant	-	-	+
6	A6	53	F	Breast	Invasive ductal carcinoma	1	IIIB	Malignant	+, 35%	-	-
7	A7	39	F	Breast	Invasive ductal carcinoma	1	IIA	Malignant	+, 8%	+++, 15%	+++
8	A8	58	F	Breast	Invasive ductal carcinoma	1	IIB	Malignant	-	-	+++
9	A9	31	F	Breast	Invasive ductal carcinoma	1	IIA	Malignant	++, 85%	++, 40%	-
10	A10	50	F	Breast	Invasive ductal carcinoma	1	IV	Malignant	-	+, 10%	-
11	A11	50	F	Breast	Invasive ductal carcinoma	1	IIIB	Malignant	-	-	-
12	A12	46	F	Breast	Invasive ductal carcinoma	1	IIB	Malignant	-	-	+++
13	A13	55	F	Breast	Invasive ductal carcinoma	1	IIA	Malignant	+++, 100%	-	-
14	A14	54	F	Breast	Invasive ductal carcinoma	1	IV	Malignant	-	-	++
15	A15	39	F	Breast	Invasive ductal carcinoma	2	IIIB	Malignant	-	-	+++
16	B1	46	F	Breast	Invasive ductal carcinoma	1	I	Malignant	+, 25%	++, 60%	++
17	B2	36	F	Breast	Invasive ductal carcinoma	1	IIA	Malignant	-	-	+++
18	B3	31	F	Breast	Invasive ductal carcinoma	1	IIA	Malignant	-	-	+++
19	B4	60	F	Breast	Invasive ductal carcinoma	1	IIA	Malignant	-	-	++
20	B5	53	F	Breast	Invasive ductal carcinoma (fibrous tissue and blood vessel)	-	IIB	Malignant	+, 40%	+++, 80%	+
21	B6	53	F	Breast	Invasive ductal carcinoma	1	IIIB	Malignant	++, 40%	-	-
22	B7	39	F	Breast	Invasive ductal carcinoma	1	IIA	Malignant	-	+++, 20%	+++
23	B8	58	F	Breast	Invasive ductal carcinoma	1	IIB	Malignant	-	-	+++
24	B9	31	F	Breast	Invasive ductal carcinoma	1	IIA	Malignant	+, 80%	++, 50%	-
25	B10	50	F	Breast	Invasive ductal carcinoma	1	IV	Malignant	-	+, 8%	-
26	B11	50	F	Breast	Invasive ductal carcinoma	1	IIIB	Malignant	-	-	-
27	B12	46	F	Breast	Invasive ductal carcinoma	1	IIB	Malignant	-	-	+++
28	B13	55	F	Breast	Invasive ductal carcinoma	1	IIA	Malignant	+++, 100%	-	-
29	B14	54	F	Breast	Invasive ductal carcinoma	1	IV	Malignant	-	-	++
30	B15	39	F	Breast	Invasive ductal carcinoma	2	IIIB	Malignant	-	-	+++
31	C1	34	F	Breast	Invasive ductal carcinoma	2	IIIB	Malignant	-	-	-
32	C2	39	F	Breast	Invasive ductal carcinoma	2	IIA	Malignant	+, 90%	++, 80%	-
33	C3	59	F	Breast	Invasive ductal carcinoma	2	IIIA	Malignant	-	-	+
34	C4	42	F	Breast	Invasive ductal carcinoma	2	IIB	Malignant	-	-	++
35	C5	47	F	Breast	Invasive ductal carcinoma	2	IIIA	Malignant	-	-	-
36	C6	48	F	Breast	Invasive ductal carcinoma	2	IIA	Malignant	+, 80%	++, 85%	+
37	C7	39	F	Breast	Invasive ductal carcinoma	2	IIA	Malignant	-	-	++
38	C8	52	F	Breast	Invasive ductal carcinoma	2	IIIB	Malignant	-	-	+++
39	C9	53	F	Breast	Invasive ductal carcinoma	2	IIA	Malignant	++, 75%	-	+++
40	C10	52	F	Breast	Invasive ductal carcinoma	2	IIIA	Malignant	+++, 100%	-	++
41	C11	51	F	Breast	Invasive ductal carcinoma	2	IIB	Malignant	-	+, 10%	+++
42	C12	47	F	Breast	Invasive ductal carcinoma	1	IIB	Malignant	-	+, 30%	+++
43	C13	50	F	Breast	Invasive ductal carcinoma	2	IIA	Malignant	+, 10%	+++, 15%	+++
44	C14	44	F	Breast	Invasive ductal carcinoma	1	IIA	Malignant	++, 80%	+++, 90%	+++
45	C15	48	F	Breast	Invasive ductal carcinoma	2	IIA	Malignant	+, 40%	+++, 90%	+++
46	D1	34	F	Breast	Invasive ductal carcinoma	2	IIIB	Malignant	-	-	-
47	D2	39	F	Breast	Invasive ductal carcinoma	2	IIA	Malignant	-	-	-
48	D3	59	F	Breast	Invasive ductal carcinoma	2	IIIA	Malignant	-	-	+++
49	D4	42	F	Breast	Invasive ductal carcinoma	2	IIB	Malignant	+, 10%	++, 10%	+
50	D5	47	F	Breast	Invasive ductal carcinoma	2	IIIA	Malignant	-	-	-
51	D6	48	F	Breast	Invasive ductal carcinoma	2	IIA	Malignant	+, 75%	++, 95%	-
52	D7	39	F	Breast	Invasive ductal carcinoma	2	IIA	Malignant	-	-	++
53	D8	52	F	Breast	Invasive ductal carcinoma	2	IIIB	Malignant	-	-	++
54	D9	53	F	Breast	Invasive ductal carcinoma	2	IIA	Malignant	+, 25%	-	+++
55	D10	52	F	Breast	Invasive ductal carcinoma	2	IIIA	Malignant	+++, 100%	++, 5%	++
56	D11	51	F	Breast	Invasive ductal carcinoma	2	IIB	Malignant	-	+, 40%	++
57	D12	47	F	Breast	Invasive ductal carcinoma	1	IIB	Malignant	-	+, 25%	++
58	D13	50	F	Breast	Invasive ductal carcinoma	2	IIA	Malignant	-	-	+++
59	D14	44	F	Breast	Invasive ductal carcinoma	1	IIA	Malignant	++, 90%	+++, 100%	+++
60	D15	48	F	Breast	Invasive ductal carcinoma	2	IIA	Malignant	+, 35%	+++, 85%	++
61	E1	64	F	Breast	Invasive ductal carcinoma	2	IIA	Malignant	-	-	+++
62	E2	45	F	Breast	Invasive ductal carcinoma	2	I	Malignant	-	-	-
63	E3	46	F	Breast	Invasive ductal carcinoma (breast tissue)	-	IIA	Malignant	-	-	+++
64	E4	47	F	Breast	Invasive ductal carcinoma	2	IIB	Malignant	-	++, 80%	-
65	E5	31	F	Breast	Invasive ductal carcinoma	2	IIB	Malignant	-	+, 10%	-
66	E6	35	F	Breast	Invasive ductal carcinoma	2	IIIB	Malignant	-	-	+++
67	E7	38	F	Breast	Invasive ductal carcinoma	2	IIB	Malignant	-	-	++
68	E8	46	F	Breast	Invasive ductal carcinoma	2	IIB	Malignant	-	-	+
69	E9	55	F	Breast	Invasive ductal carcinoma	2	IIA	Malignant	-	-	-
70	E10	38	F	Breast	Invasive ductal carcinoma	2	IIB	Malignant	-	-	+++
71	E11	70	F	Breast	Invasive ductal carcinoma	2	IIA	Malignant	-	-	-
72	E12	56	F	Breast	Invasive ductal carcinoma	2	IIB	Malignant	-	-	++
73	E13	43	F	Breast	Invasive ductal carcinoma	2	IIA	Malignant	++, 95%	+++, 95%	-
74	E14	47	F	Breast	Invasive ductal carcinoma	2	IIA	Malignant	-	-	+++
75	E15	57	F	Breast	Invasive ductal carcinoma (sparse)	-	IIA	Malignant	-	-	+
76	F1	64	F	Breast	Invasive ductal carcinoma	2	IIA	Malignant	-	-	+++
77	F2	45	F	Breast	Invasive ductal carcinoma	2	I	Malignant	-	-	-
78	F3	46	F	Breast	Invasive ductal carcinoma (breast tissue)	-	IIA	Malignant	-	++, 20%	+++
79	F4	47	F	Breast	Invasive ductal carcinoma	2	IIB	Malignant	-	+++, 95%	-
80	F5	31	F	Breast	Invasive ductal carcinoma	2	IIB	Malignant	+, 20%	-	-
81	F6	35	F	Breast	Invasive ductal carcinoma	2	IIIB	Malignant	-	-	+++
82	F7	38	F	Breast	Invasive ductal carcinoma	2	IIB	Malignant	-	-	+++
83	F8	46	F	Breast	Invasive ductal carcinoma	2	IIB	Malignant	-	-	+
84	F9	55	F	Breast	Invasive ductal carcinoma	2	IIA	Malignant	-	-	-

85	F10	38	F	Breast	Invasive ductal carcinoma	2	IIB	Malignant	-	-	+++
86	F11	70	F	Breast	Invasive ductal carcinoma	2	IIA	Malignant	-	-	++
87	F12	56	F	Breast	Invasive ductal carcinoma	2	IIB	Malignant	-	-	++
88	F13	43	F	Breast	Invasive ductal carcinoma	2	IIA	Malignant	++, 80%	++, 90%	-
89	F14	47	F	Breast	Invasive ductal carcinoma	2	IIA	Malignant	-	-	++
90	F15	57	F	Breast	Invasive ductal carcinoma	2	IIA	Malignant	-	-	-
91	G1	75	F	Breast	Invasive ductal carcinoma	2	IIB	Malignant	-	-	-
92	G2	46	F	Breast	Invasive ductal carcinoma	2	IIA	Malignant	-	-	-
93	G3	51	F	Breast	Invasive ductal carcinoma	2	IIA	Malignant	-	-	-
94	G4	36	F	Breast	Invasive ductal carcinoma	2	IIA	Malignant	++, 80%	+++, 10%	+++
95	G5	57	F	Breast	Invasive ductal carcinoma	2	IIA	Malignant	-	-	+
96	G6	52	F	Breast	Invasive ductal carcinoma	3	IIIB	Malignant	-	-	++
97	G7	66	F	Breast	Invasive ductal carcinoma	2	IIA	Malignant	-	-	-
98	G8	62	F	Breast	Invasive ductal carcinoma	1	I	Malignant	+, 35%	+, 20%	-
99	G9	53	F	Breast	Invasive ductal carcinoma	2	IIA	Malignant	+, 10%	-	+++
100	G10	40	F	Breast	Invasive ductal carcinoma	2	IIIB	Malignant	-	+, 10%	+
101	G11	37	F	Breast	Invasive ductal carcinoma	2	IIA	Malignant	-	-	++
102	G12	33	F	Breast	Invasive ductal carcinoma	2	IIA	Malignant	+, 80%	+, 40%	+++
103	G13	62	F	Breast	Invasive ductal carcinoma	3	IIIB	Malignant	-	-	++
104	G14	58	F	Breast	Invasive ductal carcinoma	2	IV	Malignant	+, 75%	-	-
105	G15	65	F	Breast	Invasive ductal carcinoma	2	IIB	Malignant	-	-	+
106	H1	75	F	Breast	Invasive ductal carcinoma	2	IIB	Malignant	+, 5%	-	-
107	H2	46	F	Breast	Invasive ductal carcinoma	2	IIA	Malignant	+, 50%	+++, 85%	-
108	H3	51	F	Breast	Invasive ductal carcinoma	2	IIA	Malignant	-	-	-
109	H4	36	F	Breast	Invasive ductal carcinoma	2	IIA	Malignant	+, 50%	+, 15%	++
110	H5	57	F	Breast	Invasive ductal carcinoma	2	IIA	Malignant	-	-	+
111	H6	52	F	Breast	Invasive ductal carcinoma	3	IIIB	Malignant	-	+, 15%	+
112	H7	66	F	Breast	Invasive ductal carcinoma	2	IIA	Malignant	-	-	-
113	H8	62	F	Breast	Invasive ductal carcinoma	1	I	Malignant	+, 40%	+, 20%	-
114	H9	53	F	Breast	Invasive ductal carcinoma	2	IIA	Malignant	-	-	+++
115	H10	40	F	Breast	Invasive ductal carcinoma	2	IIIB	Malignant	-	-	++
116	H11	37	F	Breast	Invasive ductal carcinoma	2	IIA	Malignant	-	-	++
117	H12	33	F	Breast	Invasive ductal carcinoma	2	IIA	Malignant	++, 75%	++, 35%	+++
118	H13	62	F	Breast	Invasive ductal carcinoma	3	IIIB	Malignant	-	-	++
119	H14	58	F	Breast	Invasive ductal carcinoma	2	IV	Malignant	+, 65%	+, 5%	-
120	H15	65	F	Breast	Invasive ductal carcinoma	2	IIB	Malignant	-	-	+
121	I1	54	F	Breast	Invasive ductal carcinoma	2	IIB	Malignant	-	-	++
122	I2	69	F	Breast	Invasive ductal carcinoma	2	IIIB	Malignant	-	-	-
123	I3	55	F	Breast	Invasive ductal carcinoma	2	IIIA	Malignant	-	-	-
124	I4	40	F	Breast	Invasive ductal carcinoma	2	IIA	Malignant	++, 60%	+++, 85%	-
125	I5	46	F	Breast	Invasive ductal carcinoma	2	IIB	Malignant	++, 50%	+++, 30%	+
126	I6	64	F	Breast	Invasive ductal carcinoma	2	IIB	Malignant	-	-	+++
127	I7	27	F	Breast	Invasive ductal carcinoma	2	IIB	Malignant	+, 45%	-	+++
128	I8	46	F	Breast	Invasive ductal carcinoma	3	IIA	Malignant	-	-	-
129	I9	50	F	Breast	Invasive ductal carcinoma	2	IIA	Malignant	-	-	+++
130	I10	58	F	Breast	Invasive ductal carcinoma	3	IIA	Malignant	-	-	-
131	I11	19	F	Breast	Normal breast tissue (fibrofatty tissue)	-	-	Malignant	-	*	*
132	I12	28	F	Breast	Cancer adjacent breast tissue	-	-	NAT	-	-	-
133	I13	47	F	Breast	Cancer adjacent breast tissue	-	-	NAT	-	+, 10%	-
134	I14	46	F	Breast	Cancer adjacent breast tissue	-	-	NAT	+, 30%	++, 10%	-
135	I15	42	F	Breast	Cancer adjacent breast tissue (fibrous tissue)	-	-	NAT	-	-	-
136	J1	54	F	Breast	Invasive ductal carcinoma	2	IIB	Malignant	-	-	+
137	J2	69	F	Breast	Invasive ductal carcinoma	2	IIIB	Malignant	-	-	-
138	J3	55	F	Breast	Invasive ductal carcinoma	2	IIIA	Malignant	-	-	-
139	J4	40	F	Breast	Invasive ductal carcinoma	2	IIA	Malignant	++, 80%	+++, 95%	-
140	J5	46	F	Breast	Invasive ductal carcinoma	2	IIB	Malignant	++, 60%	+++, 90%	+
141	J6	64	F	Breast	Invasive ductal carcinoma	2	IIB	Malignant	-	-	+++
142	J7	27	F	Breast	Invasive ductal carcinoma	3	IIB	Malignant	+, 50%	-	+++
143	J8	46	F	Breast	Invasive ductal carcinoma	3	IIA	Malignant	-	-	-
144	J9	50	F	Breast	Invasive ductal carcinoma	3	IIA	Malignant	-	-	++
145	J10	58	F	Breast	Invasive ductal carcinoma	3	IIA	Malignant	-	-	-
146	J11	19	F	Breast	Normal breast tissue (fibrofatty tissue)	-	-	Normal	-	-	-
147	J12	28	F	Breast	Cancer adjacent breast tissue	-	-	NAT	-	-	-
148	J13	47	F	Breast	Cancer adjacent breast tissue	-	-	NAT	-	+, 8%	-
149	J14	46	F	Breast	Cancer adjacent breast tissue	-	-	NAT	+, 20%	+, 10%	-
150	J15	42	F	Breast	Cancer adjacent breast tissue (breast ductal tissue)	-	-	NAT	-	+++, 85%	-

Supplementary Table 6. Details of image acquisition.

Samples	Cell lines	Cancer tissue	Standard RCA	smFISH
Microscope	Zeiss[#]	Zeiss[#]	Zeiss[#]	Nikon*
Magnification objectives	20 X	20 X	20 X	100 X
DAPI (Exposure time)	5-50	20-100	20-100	20
Cy7 (Exposure time)	1000	1500	1500	
FITC (Exposure time)	100	100	130	
Cy3 (Exposure time)	20-300	300-600	300	
Texas Red (Exposure time)	130	130	130	
Cy5 (Exposure time)	500	500-1000	500	1000

#, AxioplanII epifluorescence microscope from Zeiss; *, Nikon Eclipse Ti2 epifluorescence microscope.
